# Disruption of the HER3-PI3K-mTOR oncogenic signaling axis and PD-1 blockade as a multimodal precision immunotherapy in head and neck cancer

**DOI:** 10.1038/s41467-021-22619-w

**Published:** 2021-04-22

**Authors:** Zhiyong Wang, Yusuke Goto, Michael M. Allevato, Victoria H. Wu, Robert Saddawi-Konefka, Mara Gilardi, Diego Alvarado, Bryan S. Yung, Aoife O’Farrell, Alfredo A. Molinolo, Umamaheswar Duvvuri, Jennifer R. Grandis, Joseph A. Califano, Ezra E. W. Cohen, J. Silvio Gutkind

**Affiliations:** 1 Moores Cancer Center, University of California San Diego, La Jolla, CA USA; 2 Department of Pharmacology, University of California San Diego, La Jolla, CA USA; 3 Department of Surgery, Division of Otolaryngology-Head and Neck Surgery, UC San Diego School of Medicine, San Diego, CA USA; 4 Celldex Therapeutics, Hampton, NJ USA; 5 Department of Otolaryngology, University of Pittsburgh School of Medicine, Pittsburgh, PA USA; 6 Department of Otolaryngology–Head and Neck Surgery, University of California San Francisco, San Francisco, CA USA

**Keywords:** Oral cancer, Cell signalling

## Abstract

Immune checkpoint blockade (ICB) therapy has revolutionized head and neck squamous cell carcinoma (HNSCC) treatment, but <20% of patients achieve durable responses. Persistent activation of the PI3K/AKT/mTOR signaling circuitry represents a key oncogenic driver in HNSCC; however, the potential immunosuppressive effects of PI3K/AKT/mTOR inhibitors may limit the benefit of their combination with ICB. Here we employ an unbiased kinome-wide siRNA screen to reveal that HER3, is essential for the proliferation of most HNSCC cells that do not harbor *PIK3CA* mutations. Indeed, we find that persistent tyrosine phosphorylation of HER3 and PI3K recruitment underlies aberrant PI3K/AKT/mTOR signaling in *PIK3CA* wild type HNSCCs. Remarkably, antibody-mediated HER3 blockade exerts a potent anti-tumor effect by suppressing HER3-PI3K-AKT-mTOR oncogenic signaling and concomitantly reversing the immune suppressive tumor microenvironment. Ultimately, we show that HER3 inhibition and PD-1 blockade may provide a multimodal precision immunotherapeutic approach for *PIK3CA* wild type HNSCC, aimed at achieving durable cancer remission.

## Abstract (web summary)

There is an unmet need to improve the response to immune checkpoint blockade in patients with head and neck squamous cell carcinoma (HNSCC). Here the authors show that aberrant HER3 activation sustains the proliferation of PIK3CA wild type HNSCC cells and that HER3 inhibition increases response to PD-1 blockade in HNSCC preclinical models.

## Introduction

Head and neck squamous cell carcinomas (HNSCC), which arise in the oral cavity, oropharynx, larynx, and hypopharynx, remain a major public health concern. Each year approximately 600,000 new cases of HNSCC are diagnosed worldwide, ranking sixth overall in incidence^1^. In the United States alone, over 65,600 new cases of HNSCC are predicted to have occurred in 2020, resulting in 14,500 deaths^1^. The main risk factors include tobacco and alcohol consumption, and human papillomavirus (HPV) infection^2^. Despite advances in curative intent therapy over the last three decades, long-term toxicity continues to be unacceptable for many patients who are cured (up to 30–60%) while those that develop recurrent disease will inevitably succumb^3^. HNSCC patients have a 5-year survival rate of 63%, and patients with who present with locally-advanced disease face higher rates of mortality (<50% 5-year survival)^4,5^. Therefore, HNSCC represents a significant worldwide health problem with high mortality and morbidity^1^. There is an urgent need to develop new approaches to achieve durable cure and relapse-free survival for HNSCC^3^.

A striking finding from the recent deep sequencing of the HNSCC genomic landscape was the remarkable multiplicity and diversity of genetic alterations in this malignancy, most of which, nonetheless, converge into only a handful of network alterations^6,7^. These include those regulated by the *TP53*, *FAT1*, *NOTCH1*, *CASP8*, *CDKN2A* (*p16*^INK4A^) genes, and *PIK3CA* mutations^6–8^. Among them, *PIK3CA*, encoding the PI3Kα catalytic subunit, is the most commonly mutated oncogene in HNSCC (~20%), with a significant enrichment of *PIK3CA* mutations in HPV+ tumors (25%)^7,8^. In prior studies, our team contributed the early discovery that the persistent activation of the PI3K/AKT/mTOR signaling circuitry is the most frequent dysregulated signaling pathway in HNSCC (>80% of all HPV− and HPV+ cases)^9,10^. We also showed that mTOR inhibitors (mTORi) exert potent antitumor activity in multiple experimental HNSCC model systems (reviewed in^11^) and in a recent Phase 2 clinical trial in HNSCC patients^12^.

Recent immunotherapeutic strategies, such as immune check point blockade (ICB) with pembrolizumab or nivolumab (anti-PD-1), demonstrated immunomodulation and durable remissions and gained FDA’s approval in HNSCC^13,14^. However, less than 20% of HNSCC patients benefit from anti-PD-1 treatment, often failing to achieve durable response^13,15^. There is a clear need to identify therapeutic options to enhance the response to ICB in HNSCC. In this regard, how oncogenic pathways promote the evasion of tumor immune surveillance is still poorly understood^16^. This prevents the development of effective combination therapies targeting tumor driving and immune evasive mechanisms, concomitant with anti-PD-1 ICB to reinvigorate T-cell mediated tumor elimination.

In addition to the PI3K/AKT/mTOR pathway representing a major driver in HNSCC and many other cancers, PI3K and mTOR can play fundamental functional roles in the innate and adaptive immune system^17,18^. Thus, the potential immunosuppressive effects of PI3K and mTOR inhibitors may limit the benefit of their combination with immune oncology (IO) agents. A multitude of upstream components regulating the PI3K/AKT/mTOR pathway are altered in human cancers^18^, thus we reasoned that the identification of the mechanisms sustaining PI3K/AKT/mTOR signaling in >80% of HNSCC that do not harbor *PIK3CA* mutations may provide opportunities for novel combination treatment options with ICB for the majority of patients that do not respond to anti-PD-1 treatment.

Here, we employ an unbiased kinome-wide siRNA screen in *PIK3CA* wild type HNSCC cells to discover that the *ERBB3* gene, encoding HER3, is required for HNSCC proliferation and persistent AKT/mTOR signaling. By leveraging genetically-defined human HNSCC xenografts and recently developed syngeneic HNSCC mouse models, we demonstrate that co-targeting HER3 and PD-1 results in tumor growth suppression and a concomitant, enhanced therapeutic immune response, collectively resulting in durable tumor eradication.

## Results

### Growth promoting signaling by HER3 in HNSCC and limited expression in T cells

**Fig. 1:**
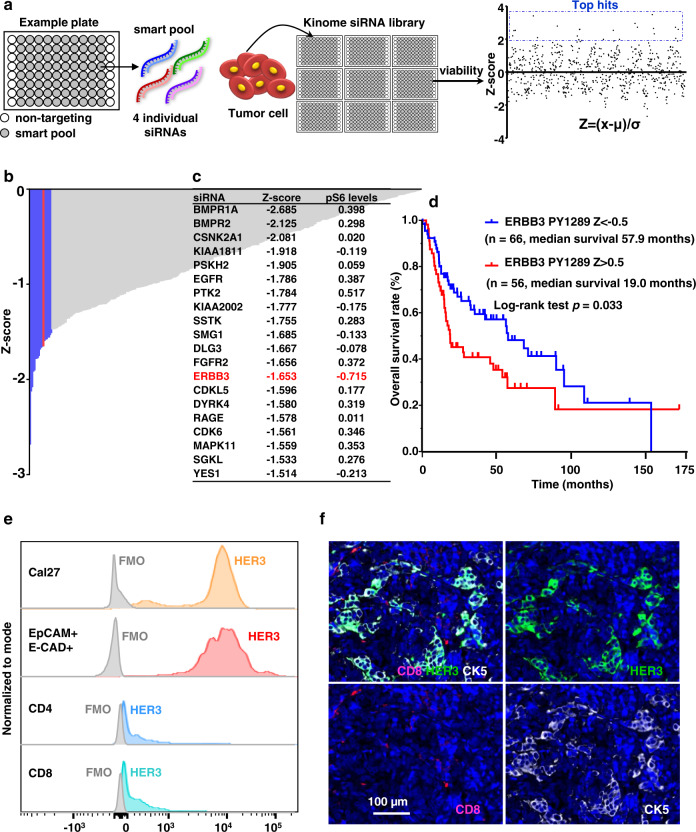
HER3 is a candidate driver of the PI3K/mTOR oncogenic signaling circuitry in HNSCC. **a** Experimental scheme of the kinome siRNA library screen. A “smart pool” of four individual siRNAs targeting each protein kinase gene of the human kinome is distributed in each well of the experimental plates. Cal27 cells were incubated for 72 h and assayed for cell viability, and the *Z*-score for viability was calculated (*Z* = (x − µ)/σ) (x stands for each value of cell viability; µ stands for average value; σ stands for standard deviation). **b** siRNA library targeting human kinases using the kinome siRNA library with Cal27 cells was conducted to search for genes that affect proliferation of HNSCC. Shown are the genes whose knockdown decrease cell viability (*Z*-score). The blue are the top 20 genes and HER3 is shown in red. (See Supplementary Data 1 for complete list). **c** Cal27 cells were transfected with the corresponding siRNAs (top 20 hits) for 72 h and cell lysates were analyzed for pS6 by western blotting. Densitometry analysis of western blots was performed using ImageJ. Shown are the top 20 hits of the kinome siRNA screen and their *Z*-scores, together with changes in pS6 levels after each gene was knocked down, as compared to the non-targeting siRNA group. Similar levels of S6 and GAPDH as loading control were confirmed (Supplementary Fig. 1). **d** The TCGA (The Cancer Genome Atlas) database was used to determine the relationship between HER3 phosphorylated on tyrosine 1289 (PY1289) and overall survival (OS) (*n* = 122 HNSCC patients, two sided log-rank test; *p* = 0.033). **e** Histogram demonstrating HER3 expression in EpCAM^+^ E-CAD^+^ tumor cells (red) and tumor-infiltrating CD4^+^ and CD8^+^ T cells (blue) in a fresh surgical specimen from a Stage II T2N0M0 primary tongue squamous cell carcinoma, compared to Cal27 cells (orange) as control. FMO (fluorescence minus one) samples were used to create HER3 + staining gates. **f** Immunofluorescent staining of CK5, CD8, and HER3 in the same specimen in panel (**e**), showing HER3 co-expressed with cancer cells (CK5 positive), but not with CD8^+^ T cells (*n* = 1 patient). Source data are provided as a Source Data file.

The human kinome contains 518 protein kinases that represent one of the most important drug targets^19^. In search for the underlying mechanisms sustaining elevated PI3K-AKT-mTOR activity in HNSCC cells that do not harbor *PIK3CA* mutations, we took advantage of the fact that signaling inhibitors are growth suppressive in HNSCC to conduct a kinome-wide siRNA screen in a *PIK3CA* wild type HNSCC cell line (Fig. 1a). This cell viability screen revealed multiple kinases whose knockdown (KD) decreased HNSCC cell proliferation (Fig. 1b). The potential function of these candidate kinases controlling HNSCC growth warrant further investigation. Of interest, the *ERBB3* gene, encoding HER3, was among the top 20 screen hits (Fig. 1c, middle column). We then conducted a counter screen analysis of these top growth suppressive hits for their ability to reduce the phosphorylated form of ribosomal protein S6 (pS6), a downstream target of mTOR that reflects mTOR pathway activation. This secondary screen revealed that *ERBB3* was the gene whose KD results in the highest reduction of pS6 (Fig. 1c right column, and see individual results in Supplementary Fig. 1). The latter finding was extended to multiple other HNSCC cellular systems (see below).

Several studies indicated that HNSCC expresses the highest levels of the HER3 ligand neuoregulin 1 (NRG) among all cancer types, correlating with poor prognosis, thereby activating HER3 in an autocrine fashion^20^. Aligned with these observations, our analysis of tyrosine phosphorylated HER3 (pHER3, HER3 phosphorylated in Tyr1289) in the HNSCC TCGA dataset demonstrated a significantly lower overall survival (OS) in patients harboring high levels of pHER3 (Fig. 1d), suggesting pHER3 was a strong predictor of poor prognosis.

To determine the differences in expression level of HER3 in cancer cells and immune cells in the tumor microenvironment, we performed flow cytometry analysis using HNSCC patient samples. Interestingly, HER3 is highly expressed in cancer cells (CD45^−^/EpCAM^+^/E-CAD^+^), but limitedly in T cells (CD45^+^/CD8^+^/CD4^+^) (Fig. 1e). Similarly, immunofluorescence staining showed co-expression of cytokeratin 5 (SCC cells) and HER3. Of importance, HER3 is not detected in T cells, suggesting that HER3 may stimulate cancer-driving mTOR activity in HNSCC cells without modulating T cell function directly (Fig. 1e, f).

### Persistent tyrosine phosphorylation of HER3 underlies aberrant PI3K/AKT/mTOR signaling in HNSCC harboring wild type *PIK3CA*

**Fig. 2:**
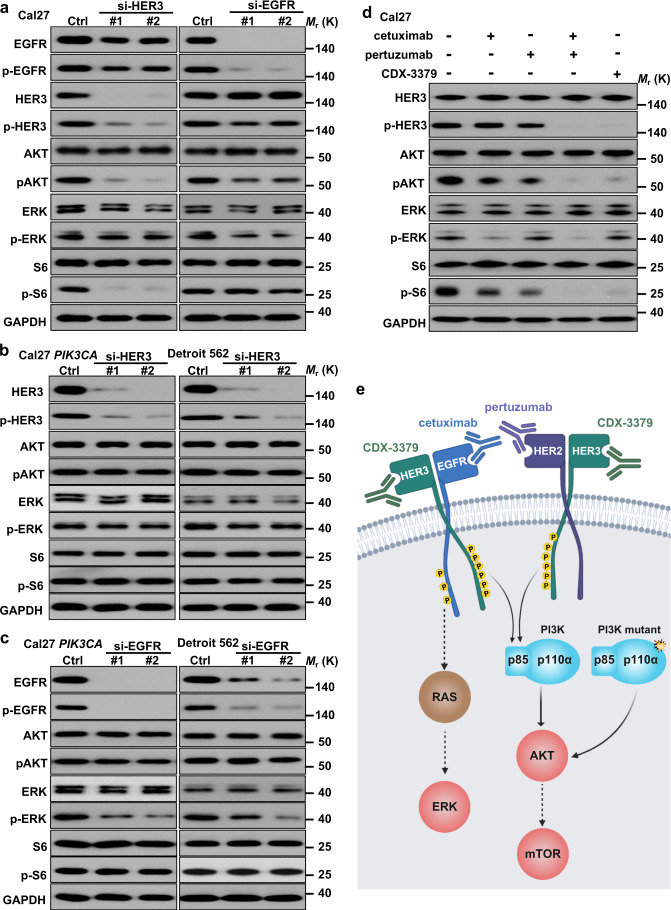
HER3 as a signaling hub stimulating the PI3K signaling circuitry in HNSCC. **a** Western blot analysis of signaling events in HNSCC cells after knock down (KD) of *ERBB3* or *EGFR*. Cal27 cells were transfected with the *ERBB3* or *EGFR* siRNAs for 72 h and cell lysates were analyzed as indicated. **b** Western blot analysis of signaling events in HNSCC cells expressing *PIK3CA* H1047R mutation after KD of *ERBB3*. Cal27 cells expressing *PIK3CA* H1047R ectopically or Detroit 562 endogenously were transfected with the *ERBB3* siRNAs for 72 h and cell lysates were analyzed by western blotting. **c** Western blot analysis of signaling events in HNSCC expressing *PIK3CA* H1047R mutation after KD of *EGFR*. Cal27 cells expressing *PIK3CA* H1047R or Detroit 562 were transfected with the *EGFR* siRNAs for 72 h and cell lysates were analyzed as indicated. **d** Western blot analysis of signaling events in HNSCC cells treated with cetuximab, pertuzumab, and CDX-3379. Cal27 cells were serum starved overnight and treated by cetuximab, pertuzumab, cetuximab + pertuzumab, and CDX-3379 at 100 ng/ml for 2 h. Cell lysates were analyzed as indicated. **e** Scheme depicting EGFR-HER3 dimer resulting in HER3 tyrosine phosphorylation and PI3K signaling stimulation by HER3-p85 interaction. Created with BioRender.com. Data shown were representative blots of results from three independent experiments with similar results. Source data are provided as a Source Data file.

HER3 is one of the ErbB family of proteins including HER1 (EGFR, ErbB1), HER2 (Neu, ErbB2), HER3 (ErbB3), and HER4 (ErbB4)^21^. HER3 lacks intrinsic kinase activity, and its phosphorylation and function is dependent on forming complexes with other catalytically active kinases, most often of the ErbB family. Indeed, ErbB proteins are capable of forming homodimers, heterodimers, and possibly higher-order oligomers upon activation by a subset of potential growth factor ligands^21^. To begin investigating the relative contribution of ErbB family dimers to the regulation of ERK and mTOR signaling in HNSCC, we knocked down EGFR (HER1) or HER3 in HNSCC Cal27 cells. Remarkably, HER3 KD specifically reduced activation of AKT and mTOR (as judged by the accumulation of pAKT and pS6) but not ERK (pERK) in this and other *PIK3CA* wild type HNSCC cell lines, including HN12 and SCC47, the latter a representative HPV+ cellular systems^22^ (Fig. 2a and Supplementary Fig. 2). However, expression of activating *PIK3CA* mutants endogenously in Detroit 562 HNSCC cells or ectopically in Cal27 cells reverted this activity (Fig. 2b), which was extended to other HNSCC cells (HN12 and SCC47) expressing *PIK3CA* mutants (Supplementary Fig. 2). In contrast, EGFR KD reduced ERK but had a limited impact on AKT and mTOR activity in all *PIK3CA* wild type cells tested (Fig. 2a, c and Supplementary Fig. 2). The selectivity of HER3 signaling to PI3K/AKT/mTOR was further investigated by the analysis of protein–protein interactions between HER3 and EGFR with p85/p110α, the regulatory and catalytic subunits of PI3K, respectively. Immunoprecipitation (IP) by HER3 showed direct association between endogenous HER3 and PI3K subunits under basal conditions, which was abolished by HER3 KD (si-HER3). In contrast, we detected weak binding of EGFR to p85/p110α, using EGFR KD (si-EGFR) as control (Supplementary Fig. 4).

Of interest, we noticed that KD of HER3 slightly reduced the total and phosphorylated forms of EGFR, while KD of EGFR had only a limited impact on total HER3 and pHER3 (Fig. 2a). This suggests that the HER3-EGFR dimer formation may help stabilize EGFR, but that EGFR activation is not solely responsible for HER3 phosphorylation. This observation was further extended using cetuximab, a monoclonal blocking antibody against EGFR that is clinically approved for HNSCC treatment^23^. Paralleling the effects of EGFR KD, cetuximab blocked pERK but reduced slightly pHER3, pAKT, and pS6 (Fig. 2d and Supplementary Fig. 3). This observation promoted us to explore whether HER2, another ErbB family member activating HER3 in multiple cancer contexts^24^, contributes to HER3 phosphorylation in HNSCC. Pertuzumab, a monoclonal antibody blocking HER2 homo- and hetero-dimerization that is approved for multiple cancer types^25^ alone did not affect pERK, but diminished slightly pHER3, pAKT, and pS6 (Fig. 2d and Supplementary Fig. 3). Remarkably, the combination of cetuximab and pertuzumab robustly decreased pHER3, and its downstream AKT and mTOR activation, to an extent similar to that achieved by the use of CDX-3379, a blocking antibody targeting HER3 directly that is in clinical development^26^. These findings suggest that heterodimers of EGFR/HER3 and HER2/HER3 promote HER3 phosphorylation, and the consequent activation of PI3K/AKT/mTOR activity by the assembly of HER3-PI3K molecular complexes, thus representing a key signaling hub in HNSCC (scheme in Fig. 2e).

### HER3 is a therapeutic target in HNSCC: Potent inhibition by CDX-3379, a HER3 blocking antibody

**Fig. 3:**
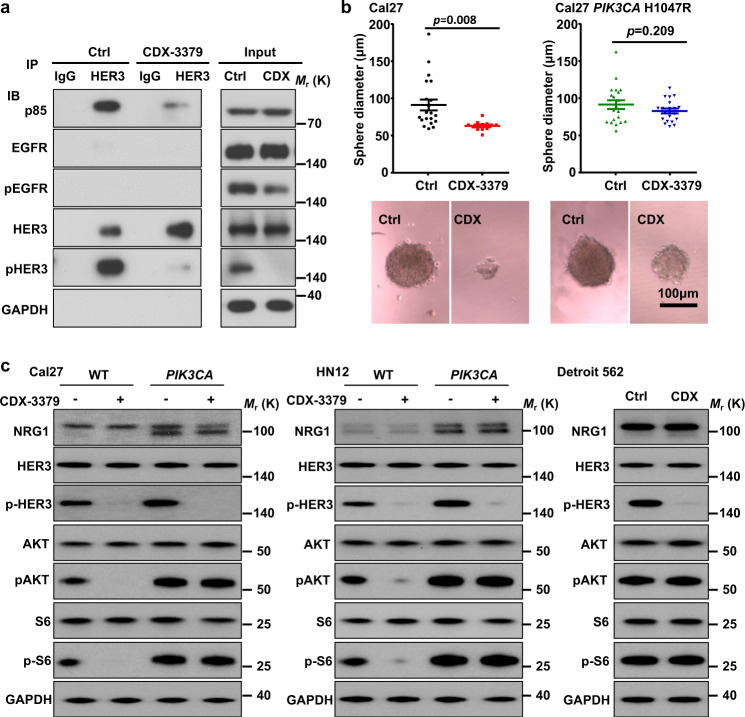
Anti-tumor effect of HER3 kinase inhibition with CDX-3379 antibody: Disruption of HER3-p85 interaction. **a** HER3 co-IP analysis of signaling events in HNSCC cells treated by CDX-3379, a HER3 blocking antibody. Cal27 cells were treated by CDX-3379 (1 µg/ml) for 1 h and cell lysates were analyzed as indicated. **b** Cal27 cells and cells expressing *PIK3CA* H1047R were plated in 96-well ultra-low attachment culture plates at 100 cells per well (*n* = 20), and treated with PBS or CDX-3379 (1 μg/ml). The diameters of sphere colonies on each well were monitored using light microscopy. Shown are diameters (top) and representative photographs (bottom) of sphere colonies of each group. (Cal27 control, *n* = 20; Cal27 CDX-3379, *n* = 12; Cal27 *PIK3CA* H1047R control, *n* = 20; Cal27 *PIK3CA* H1047R CDX3379, *n* = 20;). Data were reported as mean ± SEM; two-sided Student’s *t*-test. **c** Western blot analysis of signaling events in HNSCC cells treated by CDX-3379. Wild-type and cells infected with *PIK3CA* H1047R (*PIK3CA*) of (left) Cal27, (middle) HN12, together with (right) Detroit 562 cells (harboring *PIK3CA* H1047R mutations) were serum starved overnight and treated by CDX-3379 at 100 ng/ml for 2 h. Cell lysates were analyzed as indicated. For both Fig. a and c, data shown are representative blots of results from three independent experiments with similar results. Source data are provided as a Source Data file.

HER3 acts as a key upstream activator of mTOR, we asked whether blockade of HER3 is sufficient to influence mTOR activity and its tumor promoting effects in HNSCC. We first confirmed that CDX-3379, which decreases pHER3 levels (see above, Figs. 2d and  3a), also disrupts the association between HER3 and the p85 regulatory subunit (Fig. 3a), in a *PIK3CA* wild type HNSCC cell line (Cal27). We next investigated whether mutation of *PIK3CA* is sufficient to confer AKT-mTOR signaling and tumor resistance to CDX-3379. *PIK3CA* wild type Cal27 cells were very sensitive to the growth suppressive effect of CDX-3379 in vitro. However, Cal27 cells became resistant after expression of a typical kinase domain canonical mutation: *PIK3CA* H1047R mutant (Fig. 3b). This can be explained by the fact that *PIK3CA* wild type HNSCC cells (Cal27, HN12, and SCC47) ectopically expressing mutant *PIK3CA* have higher levels of basal AKT and mTOR activation, therefore becoming insensitive to HER3 blockade, similar to that of Detroit 562 cells, a *PIK3CA* + HNSCC cell line harboring *PIK3CA* H1047R mutations endogenously (Fig. 3c and Supplementary Fig. 5). Of interest, the levels of neuregulin (NRG1) expression in *PIK3CA* mutant Cal27 and HN12 is higher, as compared to parental cells (Fig. 3c), but not in SCC47, an HPV(+) HNSCC cell line, which has high basal levels of NRG1 expression (Supplementary Fig. 5).

**Fig. 4:**
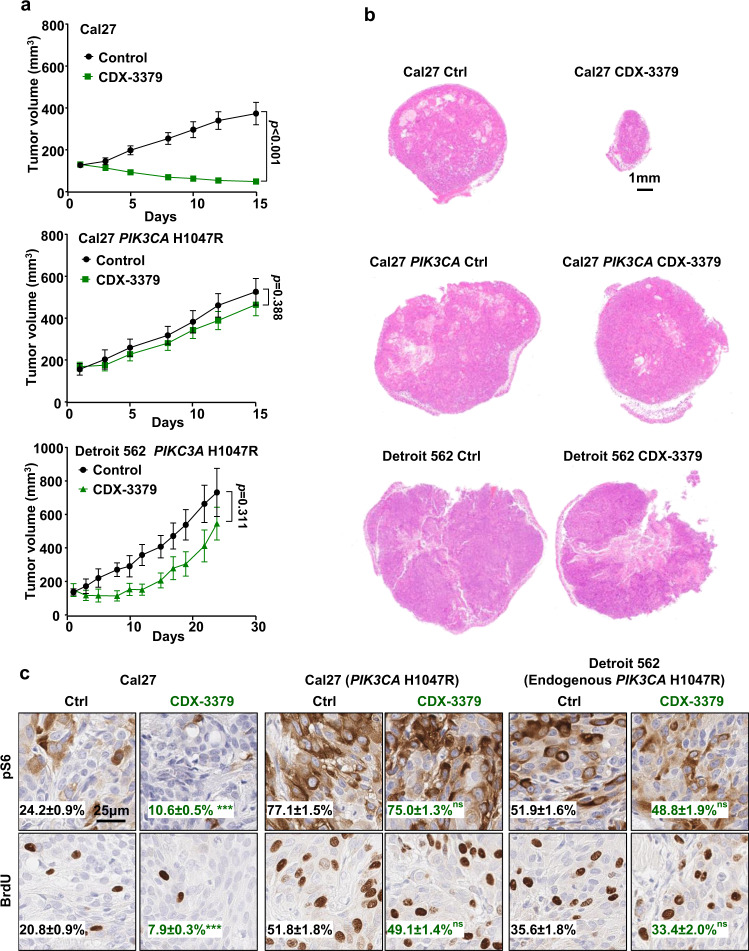
Anti-tumor effect of HER3 kinase inhibition with CDX-3379 antibody in vivo. **a** WT Cal27 cells, Cal27 cells expressing *PIK3CA* H1047R or Detroit 562 were transplanted into the flanks of athymic nude mice, and when they reached 150–200 mm^3^, mice were treated with vehicle diluent or CDX-3379 (10 mg/kg, three times/week) for the indicated days (*n* = 10 for Cal27; *n* = 10 for Cal27 *PIK3CA* H1047R; *n* = 6 for Detroit 562). Data were reported as mean ± SEM; two-sided Student’s *t*-test. **b** Representative H&E stains of mouse tumors from the experiment from panel **a**. **c** Representative immunohistochemical analysis of pS6 and BrdU in the short-term treatment (every other day for three times) groups from panel (**a**) (*n* = 4 mice per group). Brown chromogen deposition reflects the immunoreactivity; hematoxylin was used as a nuclear counterstain (blue). Scale bars represent 25 μm. Quantification from images using Qupath software and the percentage of positive staining are shown on each image. Data were reported as mean ± SEM, two-sided Student’s *t*-test, *p* > 0.05, non-significant or ns; ***p < .001 when compared with the control-treated group. Source data are provided as a Source Data file.

CDX-3379 exerts potent antitumor activity in *PIK3CA* wild type HNSCC cells in vivo (Fig. 4a, b and Supplementary Fig. 6), similar to that previously reported in multiple HPV^−^ and HPV+HNSCC cells^20,27,28^. Supporting the key role of PI3K downstream of HER3, *PIK3CA* mutations are sufficient to induce resistance to CDX-3379 (Fig. 4a, b and Supplementary Fig. 6). Aligned with the tumor growth kinetics, immunostaining (immunohistochemistry, (IHC)) showed that in *PIK3CA* wild type Cal27 cells, inhibition of HER3 by CDX-3379 decreased pS6, which correlated with decreased proliferation as depicted by BrdU incorporation. Basal pS6 and BrdU levels were increased in *PIK3CA*-mutated tumors, but CDX-3379 had a more limited inhibitory effect (Fig. 4c). As an additional experimental approach, we used *PTEN* knockout Cal27 cells, which exhibit elevated basal AKT and mTOR signaling^29^, and were resistant to CDX-3379 as shown by the failure to reduce pAKT, pS6, and tumor growth (Supplementary Fig. 7). Collectively, this supports that PI3K-AKT–mTOR may represent a key target downstream from HER3, and that CDX-3379 exerts therapeutic effects primarily in *PIK3CA* wild type HNSCC xenografts.

### HER3 inhibition in HNSCC syngeneic model: A unique opportunity to disable mTOR oncogenic signaling in HNSCC bypassing the immunosuppressive effects of mTORi

As HER3 has limited expression in immune cells (see above), we next asked whether the ability to reduce cancer-driving mTOR activity exclusively in HNSCC cells by anti-HER3 would enable us to elucidate the mechanisms by which aberrant PI3K/mTOR establishes an immune evasive tumor immune microenvironment (TIME). For these studies, we took advantage of 4MOSC1, our recently developed murine carcinogen-induced HNSCC mouse model, which is *Pik3ca* wild type and has nearly identical HNSCC tobacco-associated mutational signatures and genomic aberrations^30^. Moreover, the availability of CDX-3379, the first HER3 blocking antibody that recognizes both human and mouse HER3^26^ enabled us to investigate the direct impact of blocking HER3-PI3K/mTOR signaling in cancer cells on their TIME in syngeneic animal models.

**Fig. 5:**
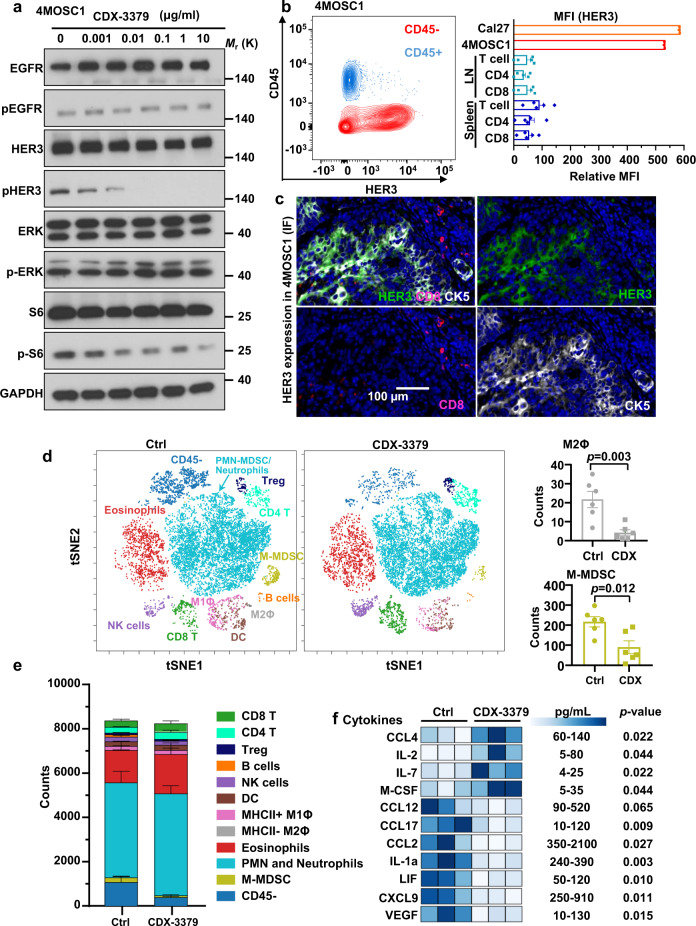
HER3 inhibition with CDX-3379 in syngeneic HNSCC model remodels the tumor immune microenvironment. **a** 4MOSC1 cells were treated with different concentrations of CDX-3379 for 1 h and cell lysates were analyzed by western blot analysis as indicated. Data shown are representative blots of results from three independent experiments with similar results. **b** Left, single cell suspension of 4MOSC1 tumors was stained with CD45 and HER3 antibodies and analyzed by flow cytometry. Shown is a representative flow cytometry plot of the frequency of tumor cells (CD45^−^) and immune cells (CD45^+^) expressing HER3 (*n* = 4). Right, in mice with 4MOSC1 tumors, HER3 expression was measured in CD90.2^+^ T cells, CD90.2^+^ CD4^+^ T, and CD90.2^+^ CD8^+^ T cells in both the cervical lymph node (LN) and splenic compartment (the relative HER3 MFI of Cal27 and 4MOSC1 cells from culture serve as a positive control). Data were reported as relative mean fluorescence intensity (MFI) as compared to FMO (*n* = 3 for Cal27 and 4MOSC1; *n* = 5 for lymph nodes and spleen); Relative MFI was calculated by subtracting MFI-HER3 from MFI-FMO. **c** Immunofluorescent staining of CD8, HER3, and CK5 in slides of 4MOSC1 tumors to show HER3 co-expressed with cancer cells (CK5 positive), but not with CD8^+^ T cells. **d** Left, C57Bl/6 mice with 4MOSC1 tumors were treated IP with isotype control antibody and CDX-3379 (20 mg/kg) (*n* = 6). Biological populations identified via CyTOF were categorized in tSNE map. FlowSom depiction of different cell lineages within in the tumor micronvironment (colored) were identified by lineage-specific markers. Right, count of M2 macrophages (M2Φ) and M-MDSC cells. Data were reported as mean ± SEM; two-sided Student’s *t*-test. **e** Total count of major immune cell population within live cells from panel (**d**). **f** Chemokine and cytokine protein expression profile in 4MOSC1 tumors. Tongue tumors were established and treated as in panel **d**. Tumor lysates (total protein concentration 2 mg/ml) were used for mouse cytokine array/chemokine array analysis. Significantly altered molecules and the pg/mL range with HER3i are shown (*n* = 3). Data were reported as mean ± SEM; two-sided Student’s *t*-test. Source data are provided as a Source Data file.

We initially confirmed that CDX-3379 disrupted the association between HER3 and p85 PI3K regulatory subunit, and consequentially decreased mTOR activity in 4MOSC1 syngeneic cells (Supplementary Figs. 8a and  5a), aligned with the finding that these cells express wild type *Pik3ca*^30^. We also confirmed that 4MOSC1 tumors express high levels of HER3 in the cancer cells but barely detectable in immune cells, particularly T cells, by flow cytometry analysis (Fig. 5b) and immunofluorescence (Fig. 5c), aligned with the human tumor data (above, Fig. 1e, f). Next, we conducted a short term treatment in mice bearing tongue 4MOSC1 tumors with CDX-3379 to determine whether HER3 blockade has immunomodulatory effects. Indeed, mass cytometry analysis (CyTOF) showed that CDX-3379 treatment resulted in a significant decrease of mononuclear myeloid derived suppressor cells (M-MDSCs) and M2 macrophages, and a decrease of cancer cells (CD45^−^) (Fig. 5d, e and Supplementary Fig. 8b, c). We validated experimentally the immunosuppressive role of MDSCs by their ability to inhibit the antigen-specific T cell cytotoxicity (Supplementary Fig. 8d).

IHC for cleaved caspase-3 showed that CDX-3379 did not cause cell apoptosis in 4MOSC1 tumors, using cisplatin as a control (Supplementary Fig. 8e), suggesting that immune cell changes during CDX-3379 treatment might not be caused by promoting apoptotic and immunogenic cell death, but by immune microenvironment remodeling. Direct measurements of cytokine/chemokine levels in tumors revealed that CDX-3379 treatment led to a significant increase in the accumulation of pro-immunogenic chemokines (CCL4) and cytokines (IL-2 and IL-7), albeit we also observed a reduction in some pro-immunogenic chemokines (e.g., MIG/CXCL9) (Fig. 5f). Concomitantly, we observed a significant reduction in multiple pro-tumorigenic and highly immune suppressive cytokines (e.g., IL-1α, VEGF, and LIF) and chemokines (e.g., MCP-1/CCL2, MCP-5/CCL12, and TARC/CCL17). Many of these immune suppressive cytokines are implicated in the expansion and activation of M2 macrophages and MDSCs in cancer^31^, which may explain their reduced tumor infiltration caused by HER3 inhibition (HER3i). These observation provided a rationale for exploring the therapeutic benefit of combining CDX-3379 and other HER3 blocking agents with IO agents, such as anti-PD-1.

### HER3 inactivation increases the response to anti-PD-1 blockade in syngeneic HNSCC mouse models

**Fig. 6:**
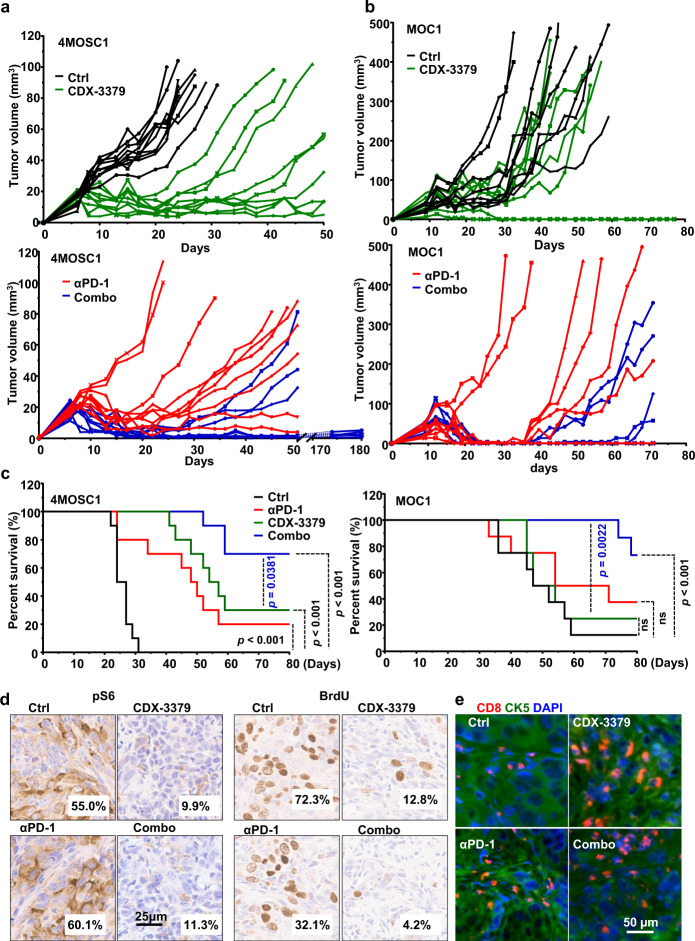
Anti-tumor effect of HER3 inhibition in syngeneic HNSCC models and increased durable responses to PD-1 blockade. **a** C57Bl/6 mice were implanted with 1 × 10^6^ of 4MOSC1 cells into the tongue. After tumors reached ~30 mm^3^, mice were treated IP with of isotype control, CDX-3379 (20 mg/kg), anti-PD-1 (10 mg/kg), or a combination of CDX-3379 and PD-1 three times per week for 3 weeks. Individual growth curves of 4MOSC1 tumor-bearing mice are shown (*n* = 10 per group). **b** C57Bl/6 mice were implanted with 2 × 10^6^ MOC1 cells into the flanks. After tumors reached approximate 50 mm^3^, mice were treated same as panel (**a**). Individual growth curves of MOC1 tumor-bearing mice are shown (*n* = 8 per group). **c** A Kaplan–Meier curve showing the survival of mice from panels (**a**) and (**b**). The death of animals occurred either naturally, when tumor compromised the animal welfare, when tongue tumor volume (panel **a**) reached 100 mm^3^ (*n* = 10 mice per group), or when flank tumor volume (panel **b**) reached 500 mm^3^ (*n* = 8 mice per group). Two sided log-rank/Mantel–Cox test. **d** Representative immunohistochemical analysis of pS6 and BrdU in the short-term treatment groups (every other day for three treatments) from panel (**a**). Brown chromogen deposition reflects the immunoreactivity; hematoxylin was used as a nuclear counterstain (blue). Scale bars represent 25 μm. Quantification from images on the left using Qupath software and the percentage of positive staining are shown on each image. **e** Immunofluorescent staining of CD8 and CK5 in the short-term treatment from panel (**a**). Source data are provided as a Source Data file.

Aligned with our previous study^30^, in 4MOSC1 model, many of the tumors initially responded to anti-PD-1 blockade, but then developed resistance over a few subsequent weeks, with only 10–20% of all tumors durably responding to anti-PD-1 blockade as defined by no regrowth upon prolonged observation of >6 months (Fig. 6a). This offered an opportunity to identify suitable therapeutic options that could sensitize tumors to anti-PD-1 in immunocompetent HNSCC experimental systems. As a single agent, CDX-3379 caused rapid tumor growth inhibition, being as potent as anti-PD-1 in terms of tumor growth delay (Fig. 6a and Supplementary Fig. 9a). However, as for nearly all targeted therapies, resistance develops and most tumor treated with HER3i relapsed and progressed after treatment termination. However, the combination of HER3 and PD-1 blockade elicited a remarkable beneficial effect, with 70% of the mice exhibiting complete and durable responses (>6 months) and, consequently, significantly increased survival (Fig. 6a, c). These findings were also confirmed using a widely-used *Pik3ca* wild type syngeneic mouse HNSCC model, MOC1 cells^32^, which also responded to the HER3/anti-PD-1 combination: mice responded to anti-PD-1 but most responders regrew after treatment termination. These cells were partially resistant to CDX-3379, likely because they harbor *Hras* mutations^32^. Albeit less potent as single agent, the combination of CDX-3379 with anti-PD-1 led to 60% of the mice achieving durable remission (Fig. 6b, c).

CDX-3379 decreased tumor pS6 staining (surrogate for mTOR activity), and tumor cell proliferation as measured by BrdU staining (Fig. 6d and Supplementary Fig. 9b). By immunofluorescence analysis we found that CDX-3379 increased CD8^+^ T cell tumor intratumoral but not peritumoral infiltration, which was not apparent when documenting the CD8^+^ T cells in the entire tumoral mass and its immediate peritumoral tissues by CyTOF (above). Similar increases were observed in the combination therapy (Fig. 6e and Supplementary Fig. 9c). Remarkably, CD8 T cell depletion reduced the response to all treatments, including anti-PD-1 and single agent CDX-3379 and its combination with anti-PD-1 (Supplementary Fig. 10). This raised the possibility that while growth suppression underlies the initial therapeutic response to HER3i, a subsequent antitumor immune response may contribute to the efficacy of HER3i in HNSCC. Indeed, CD8 T cell depletion abolished the response to anti-PD-1, as we recently reported^30^; but in the case of anti-HER3, all mice had an initial response to HER3i followed by a rapid tumor regrowth (Supplementary Fig. 10), contrasting the prolonged response achieved in most immunocompetent mice (Fig. 6a). This suggests that the initial direct blockade of HER3/PI3K/AKT/mTOR signaling in cancer cells plays a tumor inhibitory role at the beginning of CDX-3379 treatment, while the TIME remodeling activity of this HER3i may then stimulate adaptive anti-tumoral responses that are circumvented by CD8 T cell depletion. Ultimately, in the setting of *PIK3CA* wild type HNSCC the direct cancer-targeting and antitumor immune effects of CDX-3379 are augmented by concomitant anti-PD-1 blockade, leading to increased durable responses to the combination of anti-PD-1 and HER3i.

## Discussion

New precision therapeutic options are now possible to prevent and treat cancer with the elucidation of the genomic landscape of most solid tumors. In the case of HNSCC, activation of the PI3K/mTOR signaling circuitry is the most frequently dysregulated signaling pathway^6,8,11^, and this reliance on PI3K/mTOR signaling for tumor growth may in turn expose a key cancer vulnerability that can be exploited therapeutically. However, most targeted therapies activate compensatory pathways leading to drug resistance and tumor recurrence. In contrast, immunotherapies can result in prolonged or even durable responses, albeit often only a minority of the patients respond in most cancers, including HNSCC^3,13,14^. Clearly, alternative therapeutic approaches are urgently needed. Here, we identified HER3 as a druggable target to suppress aberrant PI3K/mTOR signaling in *PIK3CA* wild type HNSCC cells. Indeed, HER3 blockade with CDX-3379 promoted the regression of human HNSCC xenografts in immune deficient mice, but not in tumors that express activated *PIK3CA* mutants or in which aberrant pathway activation downstream of HER3 is sustained by *PTEN* deletion. As HER3 is not expressed in T cells, this provided a unique opportunity to disable PI3K/mTOR oncogenic signaling in HNSCC while bypassing the immunosuppressive effects of small molecule inhibitors of downstream targets in this signaling axis. Unexpectedly, we found that HER3 blockade with CDX-3379 reverses the immune suppressive tumor microenvironment, and that its antitumor activity in vivo is in part dependent on its immunomodulatory activity in addition to its concomitant growth suppressive effects by disabling a key oncogenic signaling pathway. Furthermore, we found that HER3 inactivating antibody increase the response to anti-PD-1 treatment, and that HER3 and PD-1 co-targeting results in prolonged cancer remission in most treated animals, thus revealing a promising combination treatment option for HNSCC.

A general perspective from our studies is that persistent basal HER3 tyrosine phosphorylation underlies PI3K/AKT/mTOR activation by directly binding PI3K p85/p110 subunits in most HNSCC cases that do not harbor *PIK3CA* mutations. HER3 has been primarily studied in HNSCC as a rapidly activated compensatory pathway promoting resistance to anti-EGFR therapies^33,34^. HER3 is a kinase-deficient receptor that is phosphorylated in a ligand (neuregulin-NRG/heregulin-HRG)-dependent manner through the formation of heterodimers with other HER receptors, and in HNSCC specifically by EGFR and HER2, both of which are widely expressed in this tumor type^20,21,35^, as judged by blocking antibody studies. Unlike EGFR and HER2, the intracellular domain of HER3 contains six consensus tyrosine motifs that upon phosphorylation provide high affinity binding to the p85 regulatory subunit of PI3K^36^, thereby eliciting very potent activation of the PI3K/AKT/mTOR signaling axis^21,37^. Indeed, we found that in HNSCC cells a large fraction of PI3K protein is persistently associated with HER3, but not EGFR, under basal conditions, which in turn promotes aberrant PI3K/AKT/mTOR oncogenic signaling. As such, HER3 may represent a key and underappreciated signaling hub in HNSCC. Furthermore, as both EGFR and HER2 converge to activate HER3, it may be more beneficial to inhibit HER3 directly, thereby bypassing multiple known mechanisms of horizontal resistance to EGFR and HER2 blockade. Of note, the majority of HNSCC cases do not exhibit *PIK3CA* mutations but retain mTOR hyper-activation (reviewed in^6^). Together, this provides the rationale for targeting HER3 as a precision therapeutic approach for *PIK3CA* wild type HNSCC cases.

In this context, prior studies documented the activity of targeting HER3 alone and enhanced activity when co-targeting EGFR in multiple HNSCC in genetically unselected preclinical models^20,34^. However, translation of these findings to the clinic has been challenging. Two HER3 targeting antibodies, duligotuzumab (an EGFR/HER3 bispecifc mAb) and patritumab (a HER3-targeting mAb, dosed in combination with cetuximab) failed to demonstrate benefit over cetuximab alone in respective Phase 2 trials^38,39^. While initially discouraging, review of clinical and preclinical data suggests that these agents may not have been sufficiently potent to effectively compete with its high affinity ligand NRG that is overexpressed in HNSCC and whose expression is further enhanced by elevated PI3K function, and/or dosed at sufficiently high levels to achieve meaningful target inhibition^38^. By contrast, CDX-3379 has been selected and engineered to overcome these limitations. The crystal structure of CDX-3379 binding to HER3 has been solved, which revealed that it binds HER3 outside of the NRG-ligand binding domain and locks HER3 in its auto-inhibited configuration, making the HER3 incapable of binding ligand or dimerizing with other receptors^26,40^. These findings suggest that CDX-3379 may inhibit HER3 via two distinct primary mechanisms of action, ligand-dependent (i.e., NRG-driven HER3 activation) and ligand-independent (i.e., activation driven by high activity of EGFR, HER2, or multiple other tyrosine kinases^41^). The Fc portion of CDX-3379 was engineered to enhance binding to the neonatal Fc receptor (FcRN) and thus enhancing its serum half-life in patients and target exposure. These advantages have been borne out in the clinic; CDX-3379 has a calculated serum half-life of 17 days, which combined with its potency, ensures prolonged inhibition of HER3 phosphorylation in HNSCC tumors from patients, as demonstrated in a recently completed window-of-opportunity study^42^. In addition, CDX-3379 has demonstrated clinical activity as a monotherapy (NCT02014909)^43^ and in combination with cetuximab (NCT03254927)^44^. Based on these findings, CDX-3379 may represent an anti-HER3 therapeutic mAb with improved efficacy for HNSCC.

HNSCCs deploy multiple mechanisms to avoid immune recognition and subsequent antitumor immune response, including the recruitment of MDSCs and conditioning of the surrounding microenvironment by expressing immune suppressive chemokines and cytokines, leading to the accumulation of suppressive regulatory T cells (Tregs) and the polarization of macrophages toward an immune suppressive (M2) tumor associated macrophage (TAM) phenotype^45^. The use of syngeneic HNSCC preclinical mouse models provided the first opportunity to investigate the impact of HER3i in the TIME. CDX-3379 treatment rapidly reduced multiple pro-tumorigenic and immune suppressive cytokine levels in the TIME. Further studies will be required to elucidate the precise cell populations involved and the individual role of each immune modulatory cytokine and chemokine that results in reduced infiltration of M-MDSCs and M2 macrophages, and increased intratumoral CD8 T cells. As HER3 is poorly expressed in immune cells, this reduction is likely initiated by CDX-3379 inhibition of cancer-driving PI3K-AKT-mTOR activity in HNSCC cells, which may in turn result in reduced chemokine and cytokine secretion in cancer cells directly, for example through a translational control^46^, thus disrupting a complex immune suppressive cross talk among immune cells in the TIME. Together, these findings suggest that in HNSCC overactive PI3K/mTOR activity may establish of an immunosuppressive microenvironment in addition to its best known function in growth promotion, and that these two cancer-driver processes can be concomitantly suppressed by HER3 blockade. These, and yet to be identified mechanisms, may explain the encouraging outcome of co-targeting HER3 and PD-1 in our preclinical HNSCC mouse models.

Altogether, our studies support that HER3 may represent a key signaling hub in HNSCC, as the persistent tyrosine phosphorylation of HER3 underlies aberrant PI3K/mTOR signaling in HNSCC harboring wild type *PIK3CA*. In turn, HER3 blockade with CDX-3379 may exert its antitumor effects by suppressing the HER3-PI3K-AKT-mTOR tumor promoting circuity and reversing the immune suppressive TIME. Ultimately, our findings reveal a signal transduction-based multimodal therapeutic approach for HNSCC. Our study provides a rationale for future precision immunotherapy clinical trials disrupting the PI3K/mTOR oncogenic and its associated immune evasive signaling axis in HNSCC, by targeting HER3 combined with anti-PD-1 ICB.

## Methods

### Reagents

PD-1 antibody (clone J43, #BE0033-2), isotype control antibody (#BE0091), and CD8 depletion antibody (Clone YTS 169.4, catalog #BE0117) were purchased from Bio X Cell (West Lebanon, NH). CDX-3379 was obtained from Celldex Therapeutics (Hampton, NJ). Fluorochrome-conjugated antibodies were purchased from BD Biosciences (San Jose, CA) and BioLegend (San Diego, CA). All other chemicals and reagents were from Sigma-Aldrich (St. Louis, MO) unless indicated.

### Cell lines and tissue culture

Human HNSCC cell lines Cal27, HN12, SCC47, and Detroit 562 were obtained from the NIDCR (National Institute of Dental and Craniofacial Research) cell collection^47^. The 4MOSC1 cell line was generated in-house and has been published recently^30^. MOC1 cells were generously provided by Dr. R. Uppaluri^48^. DNA authentication of cell lines was confirmed by multiplex STR profiling (Genetica DNA Laboratories, Inc. Burlington, NC) to ensure the consistency of cell identity. MycoProbe Mycoplasma Detection kit from R&D Systems (Minneapolis, MN) was used to ensure the absence of mycoplasma in cells. Cal27, HN12, SCC47, and Detroit 562 were cultured in Dulbecco’s modified Eagle’s medium supplemented with 10% fetal bovine serum and 1% antibiotic/antimycotic solution. 4MOSC1 cells were cultured in Defined Keratinocyte-SFM medium supplemented with EGF Recombinant Mouse Protein (5 ng/ml), Cholera Toxin (50 pM) and 1% antibiotic/antimycotic solution. MOC1 cells was cultured in HyClone™ Iscove’s Modified Dulbecco’s Medium (IMDM; GE Healthcare Life sciences, South Logan, UT, USA, #sh30228.02)/HyClone™ Ham’s Nutrient Mixture F12 (GE Healthcare Life sciences# sh30026.01) at a 2:1 mixture with 5% fetal bovine serum, 1% antibiotic/antimycotic solution, 5 ng/mL EGF, 400 ng/mL hydrocortisone (Sigma Aldrich, St Louis, MO, USA, #H0135), and 5 mg/mL insulin (Sigma Aldrich, #I6634). All cells were cultured at 37 °C in the presence of 5% CO_2_.

### DNA constructs, lentivirus, siRNA library, and individual siRNAs

pLVX-N-*PIK3CA* H1047R Lentiviruses were used to generate cells expressing an activated allele of *PIK3CA *upon doxycycline addition (1μg/ml in cell culture media or 625 mg/kg in mice diet). The DNA  was kindly provided by Dr. Nevan Krogan lab. Human siGENOME siRNA Library - Protein Kinases - SMARTpool (catalog number G-003505) were purchased from GE Healthcare Dharmacon Inc. siRNAs were purchased from Millipore-Sigma (Billerica, MA, USA). For Cal27 *PTEN* KO cells, lenti-CRISPR v2 plasmid was purchased from Addgene (#52961). sgRNAs targeting sequences for *PTEN* is 5′-ACCGCCAAATTTAATTGCAG-3′. This cell line was recently published by our group^29^.

### Cell viability assays

Aquabluer solution cell viability reagent was purchased from MultiTarget Pharmaceuticals LLC (Colorado Springs, CO). Cells were cultured in plates of siRNA library for three days and the viability assay was completed according to the manufacturer’s instructions.

### Immunoblot analysis and IP assay

Cells were lysed in RIPA buffer (50 mM Tris-HCl, 150 mM NaCl, 1 mM EDTA, 1% Nonidet P-40) supplemented with protease and phosphatase inhibitors (Thermofisher Scientific, #78440). Equal amounts of total proteins were subjected to SDS-polyacrylamide gel electrophoresis and transferred to PVDF membranes. Membranes were blocked 5% nonfat dry milk in TBS-T buffer [50 mmol/L Tris/HCl, pH 7.5, 150 mmol/L NaCl, 0.1% (v/v) Tween-20] for 30 min and incubated with primary antibodies in blocking buffer for 2 h at room temperature. Detection was conducted by incubating the membranes with horseradishperoxidase (HRP)-conjugated secondary antibodies (Southern Biotech) at 1:20,000 dilution for 1 h at room temperature and visualized with Immobilon Western Chemiluminescent HRP substrate (Millipore)^46,47^. For IP assay, cell lysates were incubated with Protein A Agarose Beads (Millipore-Sigma, #16-125) conjugated with antibody in 4 °C overnight. IP experiments were done according to the manufacturer’s instructions and were followed by western blot experiments. Antibodies used were from Cell Signaling Technology (Danvers, MA), pEGFR (#2234, 1:2000), NRG1 (#2573, 1:2000), pHER3 (#4791, 1:2000), p110 (#4255 S, 1:2000), pAKT^T308^ (#2965, 1:3000), pS6 (#2211, 1:5000), S6 (#2217, 1:5000), pERK (#4370, 1:2000), ERK (#9106, 1:3000), and GAPDH (# 2118, 1:8000), or Santa Cruz Biotechnology (Dallas, TX), EGFR (#sc-120, 1:2000), HER3 (#sc-7390, 1:2000), or Millipore-Sigma (Billerica, MA), p85 (# ABS234, 1:1000). The uncropped scans of blots are available as a Source Data file.

### In vivo mouse models and analysis

All the animal studies using HNSCC tumor xenografts and orthotropic implantation studies were approved by the University of California San Diego (UCSD) Institutional Animal Care and Use Committee (IACUC), with protocol ASP #S15195; and all experiemnts adhere with all relevant ethical regulations for animal testing and research. All mice were obtained from Charles River Laboratories (Worcester, MA). Mice at UCSD Moores Cancer Center are housed in individually ventilated and micro-isolator cages supplied with acidified water and fed 5053 Irradiated Picolab Rodent Diet 20. Temperature for laboratory mice in this facility is mandated to be between 18–23 °C with 40–60% humidity. The vivarium is maintained in a 12-hour light/dark cycle. All animal manipulation activities are conducted in the laminar flow hoods. All personnel are required to wear scrubs and lab coat, mask, hair net, dedicated shoes, and disposable gloves upon entering the animal rooms. For xenograft models, HNSCC cells were transplanted into both flanks (2 million per tumor) of female athymic mice (nu/nu, 4–6 weeks of age); MOC1 cells were transplanted into both flanks (2 million per tumor) of female C57Bl/6 mice (4–6 weeks of age). When average tumor volume reached a predetermined volume (150–200 mm^3^ for HNSCC xenograft and 40–60 mm^3^ for MOC1 model), the mice were randomized into groups for subsequent experiments. For orthotropic implantation, 4MOSC1 cells were transplanted (1 million per mouse) into the tongue of female C57Bl/6 mice (4–6 weeks of age). Upon tumor formation on day 5, the mice were randomized into groups. For drug treatment, the mice were treated by intraperitoneal injection (ip) with isotype control antibody, PD-1 antibody, or CDX-3379. The mice were sacrificed at the indicated time points (or when mice succumbed to disease, as determined by the ASP guidelines) and lesions were isolated for histological and immunohistochemical evaluation or flow cytometric analysis.

### Patient samples

Specimen samples were provided by Moores Cancer Center at UC San Diego Health Comprehensive Biorepository, which is funded by the National Cancer Institute (NCI P30CA23100). The BTTR policies and procedures were written in accordance with federal policy on the Protection of Human Subjects (DHHS Policy 45 CFR Part 46, FDA Policy 21 CFR Parts 50 and 56). We have obtained informed consent from all participants. The data consist of time of collection, diagnosis of the tumor stage and site, clinical outcome and other demographical information. Patient information is deidentified as much as possible and the data are safeguarded with multiple levels and layers of security. Encryption is required for all health-related personal data. Human surgical specimens were used for histological and immunofluorescence evaluation and flow cytometric analysis.

### Sphere-formation Assay

HNSCC cells were cultured in ultra-low attachment 96-well plates (Corning, Tweksbury, MA) at 100 cells per well. The medium consisted of DMEM/F12 Glutamax supplement medium (#10565042), N2 supplement (1:100 dilution, #17502-048), human recombinant EGF (20 ng/ml, #PHG0313), bFGF (20 ng/ml, #13256029), and B-27 (1:50 dilution, #17504044). All reagents were purchased from Thermo Fisher Scientific. The drug was added on the same day as seeding. Ten days post-culture, photographs were taken and the diameters of sphere colonies larger than 50 μm were measured by light microscopy.

### Immunohistochemistry and BrdU Staining

Fixation of samples was performed by zinc formalin (Z-Fix, Anatech) and tissues were paraffin-embedded; samples were sectioned into 5 µm sections and stained with Hematoxylin-Eosin. For IHC studies, samples were deparaffinized, hydrated with graded ethanols, and the endogenous peroxidase was blocked with 3% H_2_O_2_ in 70% ethanol. After washing with distilled water, antigen retrieval was performed with IHC antigen retrieval solution (Thermofisher Scientific, # 00-4955-58) in a microwave at the high setting. Slides were then washed with water and PBS, and incubated with the primary and secondary antibodies, and developed with the ABC reagent (Vector Laboratories, # PK-6100) and the DAB substrate kit (Vector Laboratories, # SK-4105)^47,49^. The following antibodies were used: pS6 (Cell Signaling Technology #2211, 1:200), Brdu (Bio-Rad #OBT0030S, 1:100), and Cleaved Caspase-3 (Cell Signaling Technology # 9661, 1:400). Samples were scanned with Axioscan Z1 (Zeiss) and analyzed using QuPath^50^ software.

### Immunofluorescence staining and analysis

Tissues were harvested, processed (see above, Immunohistochemistry and BrdU Staining section), and stained. Slides were stained for CD8 (Abcam ab22378) (1:400), HER3 (Cell signaling Technology #12708 S) (1:200), and CK5 (Fitzgerald, 20R-CP003) (1:500) antibodies. Quantification of immune infiltration was performed using QuPath, an open source quantitative Pathology & Bioimage Analysis software. At least three regions of interest (ROI) were chosen for each group and the percentage of CD8 positive cells was calculated.

### TIL isolation and flow cytometry

Tumors were isolated, minced, and re-suspended in DMEM media supplemented with 10% FBS, 1% antibiotics, and 1 mg/mL Collagenase-D (Roche, Indianapolis, IN). Tissues were incubated at 37 °C for 30 min with shaking, washed with fresh media, and passed through a 100-µm strainer to acquire a single-cell suspension. Samples were washed with PBS and processed for live/dead cell discrimination using Fixable Viability Stain 510 (BD Biosciences). Cell surface staining was done for 30 min at 4 °C with the following mouse fluorochrome-conjugated antibodies: CD45 (30-F11) (1:100), CD90.2 (30-H12) (1:200), CD8a (53-6.7) (1:100), CD4 (RM4-4) (1:400), and Ep-CAM (G8.8) (1:100); or human fluorochrome-conjugated antibodies: CD45 (HI30), CD3 (HIT3A), CD8a (HIT8A), CD4 (RPA-T4), Ep-CAM (9CA), and E-cadherin (67A4). GFP-HER3 antibody was obtained from (Celldex) and used at a (1:100) dilution for both mouse and human cells. All flow cytometry was run using BD LSRFortessa and analyzed using FlowJo. TIL count was determined using CountBright™ Absolute Counting Beads (ThermoFisher Scientific). Downstream analysis was performed using TreeStar FlowJo, version 10.6.2. A representative gating strategy for flow cytometry experiments is depicted in Supplementary Fig. 11.

### Mass cytometry

For viability staining, cells were washed in PBS and stained with Cell-ID Cisplatin (DVS Sciences) to a final concentration of 5 μM for 5 min at room temperature. Cisplatin was quenched when cells were washed and stained with the antibody cocktail. Antibodies were prepared in Maxpar cell staining buffer (PBS with 2 mM EDTA, 0.1% BSA, 0.05% NaN_3_) and incubated with cells for 15 min at room temperature. Cells were stained with the following antibodies from Fluidigm: B220 (RA3-6B2), CD117(2B8), CD11c (N418), CD25 (3C7), CD4 (RM4-5), CD45 (30-F11), CD8a (53-6.7), MHC-II (M5/114.15.2), NKP46 (29A1.4), and TCRb (H57-597); or from BioLegend: CD103 (2E7), CD115 (AFS98), CD11b (M1/70), CD19 (6D5), CD23 (B3B4), CD3 (145-2C11), CD64 (X54-5/7.1), F4/80 (BM8), FCeRI (Mar-1), FR4 (TH6), Ly6C (HK1.4), Ly6G (1A8), and NK1.1 (PK136); or from eBioscience (ThermoFisher Scientific): Siglec-F (1RNM44N). All antibodies were used at a 1:100 dilution. After staining, cells were washed and fixed with 1.6% formaldehyde (FA) for 10 min at room temperature. For cell identification, cells were washed in staining buffers and stained with DNA intercalator (Fluidigm) containing natural abundance Iridium (191Ir and 193Ir) prepared to a final concentration of 125 nM. Cells were washed with staining buffer and pelleted. Before acquiring, cells were resuspended in 0.1X dilution of EQ Four Element Calibration beads (Fluidigm) and filtered through a 35 μm nylon mesh filter. Cells were acquired on a Helios CyTOF Mass Cytometer (Fluidigm) at an event rate of 200 events/second or less. Data were normalized using Matlab-based normalization software based on the EQ bead-removal. To detect clusters of cells with a similar expression of surface markers in CyTOF, single cells were gated and clustered using unsupervised dimensionality reduction algorithm t-Distributed Stochastic Neighbor Embedding (tSNE) algorithm in Cytobank (1000 iterations, Perplexity 30, Theta 0.5, and final KL divergence was 4.926).

### Mouse chemokine array

Tumors were isolated and tissue homogenate in lysis buffer (20 mM Tris HCl pH 7.5, 0.5% Tween 20, 150 mM NaCl) supplemented with protease inhibitors. Mouse Chemokine Array 44-Plex (MD44) was run by EVE Technologies (Calgary, AB, Canada).

### MDSC inhibition of cytotoxic T cell activity

Orthotopic 4MOSC1 tongue tumors were resected before digestion with the MACS mouse tumor dissociation kit (Miltenyi Biotec, #130-096-730). MDSCs were then isolated from the tumor digest with the MDSC isolation kit (Miltenyi Biotec, #130-094-538), through which enriched Gr-1^high^Ly-6G^+^ (PMN-MDSC) and Gr-1^dim^Ly-6G^−^ (M-MDSC) populations were isolated. OT-1 splenocytes were isolated from OT-1 mice expressing anti-ovalbumin (OVA) specific T cell receptor^51^ through mechanical separation and red blood cell lysis. After isolation of cell populations, splenocytes were cultured for three days with CD3 and CD28 antibodies, and either 5% PMN-MDSCs, 5% M-MDSCs, or control condition with no MDSCs. Melanoma cells expressing the ovalbumin antigen (B16-OVA) cells were plated in a 96 well plate at 5000 cells/well, and splenocytes from the above conditions were added at 50,000 cells/well. Additional control conditions of B16-OVA cells + 10% DMSO, as well as B16-OVA alone, were also plated. After 48 h of coculture, the wells were evacuated and washed to remove splenocytes in suspension, and the AquaBluer redox indicator was added (MultiTarget, # 6001). Data were gathered from the plate 48 h after coculture, and percent relative fluorescence units (%RFU) was calculated as the percent difference of the data from the average fluorescence value of the B16-OVA control samples; the ratio represents % viability.

### The Cancer Genome Atlas (TCGA) analysis

The TCGA database was used to determine the relationship between ERBB3 (HER3) PY1289, and OS. Multi-omics data of ERBB3 PY1289 and clinical information (TCGA-HNSCC, provisional) was downloaded from the cBio Portal (http://www.cbioportal.org/).

### Statistics and reproducibility

Data analysis were performed with GraphPad Prism version 7 for Windows. The differences between experimental groups in nuclei/cell density and tumor volume were analyzed using independent *t*-tests. Survival analysis was performed using the Kaplan–Meier method and log-rank tests. The asterisks in each figure denote statistical significance, or ns for non-significant *p* > 0.05; *p < 0.05; **p < 0.01; and ***p < 0.001. All the data were reported as mean ± SEM (standard error of the mean). For all experiments, each experiment was independently repeated for at least three times with similar results.

### Reporting Summary

Further information on research design is available in the Nature Research Reporting Summary linked to this article.

## Supplementary information

Supplementary Information

Descriptions of Additional Supplementary Files

Supplementary Data 1

Reporting Summary

## Data Availability

Genomic and clinical data of HNSCC patients from TCGA were downloaded from the publicly available cBioPortal [http://www.cbioportal.org/]. Source data are available as a Source Data file. The remaining data are available within the Article, Supplementary information are available from the authors upon request. Source data are provided with this paper.

## Source data

Source Data
