# Supplementary material for: Disruption of the HER3-PI3K-mTOR oncogenic signaling axis and PD-1 blockade as a multimodal precision immunotherapy in head and neck cancer
Source: Nat Commun. 2021 Apr 22;12:2383. doi: 10.1038/s41467-021-22619-w (PMC8062674; doi:10.1038/s41467-021-22619-w)
Supplement: Supplementary file 1 — Supplementary Information [file 41467_2021_22619_MOESM1_ESM.pdf]

Supplementary information for:

Disruption of the HER3-PI3K-mTOR oncogenic signaling axis and PD-1 blockade as a multimodal precision immunotherapy in head and neck cancer

Wang *et al*.,

Supplementary Figure 1

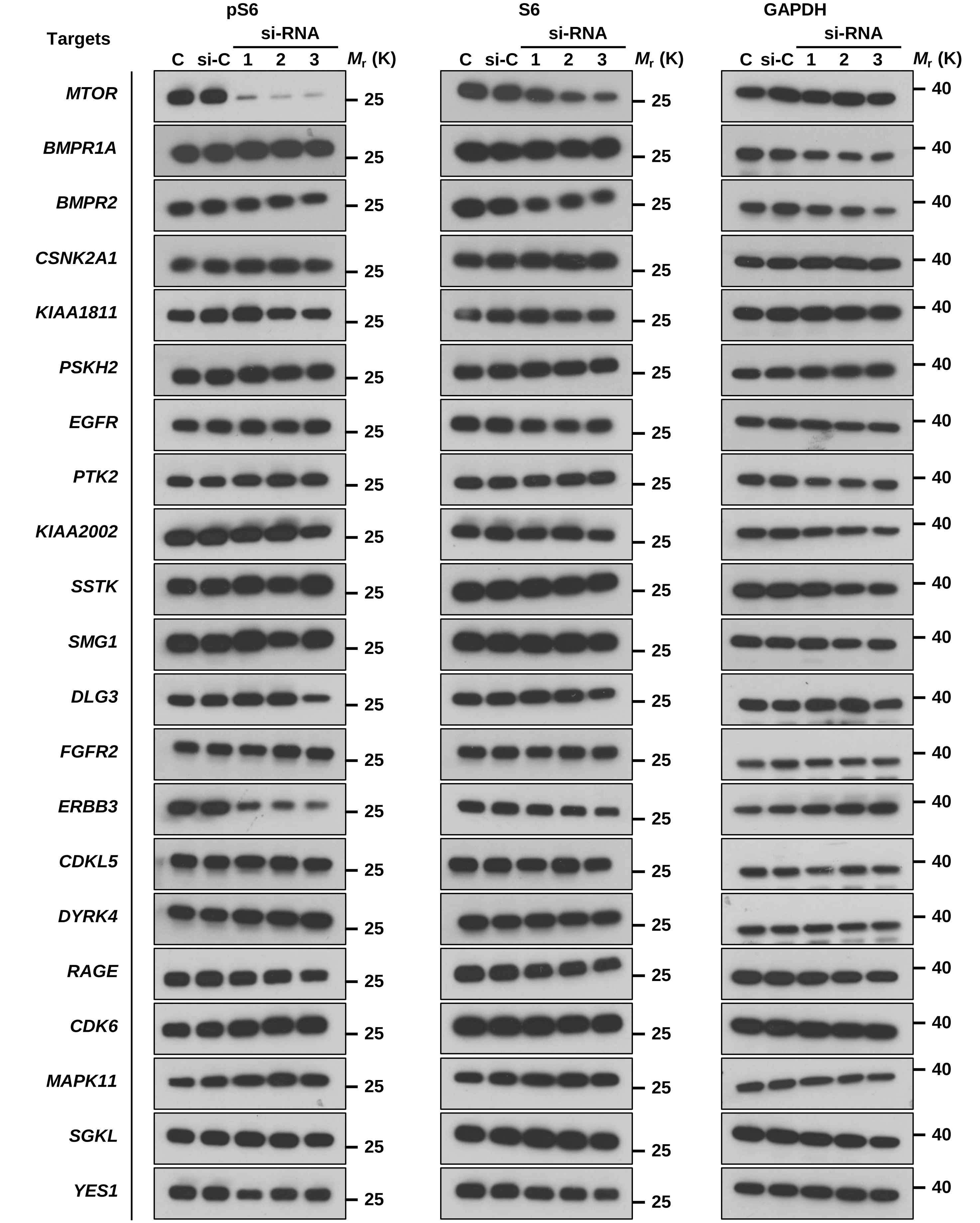

Supplementary Fig. 1. Validation of top candidates from kinome siRNA library screen in Cal27 cells. Cal27 cells were transfected with the corresponding siRNAs (top 20 hits of siRNA library, and mTOR) for 72 hours and cell lysates were analyzed for pS6, total S6 and GAPDH by western blots. Data shown are representative blots of results from 3 independent experiments with similar results. Source data are provided as a Source Data file.

Supplementary Figure 2

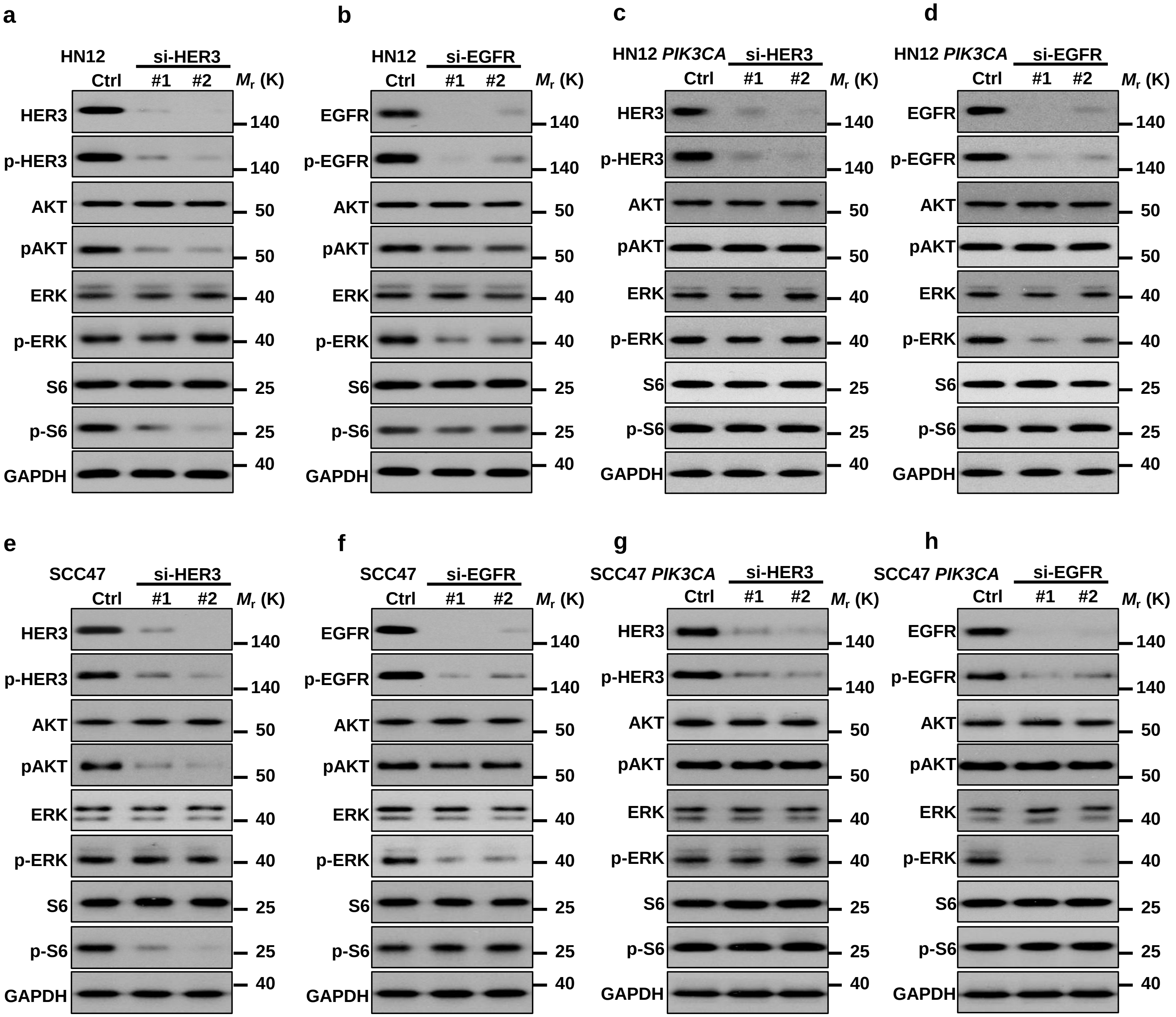

Supplementary Fig. 2. Western blot analysis of signaling events in the indicated HNSCC cells after KD of *ERBB3* or *EGFR*. (a-d) HN12 cells wild-type and infected with *PIK3CA* H1047R (*PIK3CA*) were transfected with the *ERBB3* or EGFR siRNAs for 72 hours and cell lysates were analyzed as indicated. **(e-h)** Western blot analysis of signaling events in the indicated HNSCC cells after KD of *ERBB3* or *EGFR*. SCC47 cells wild-type and infected with *PIK3CA* H1047R (*PIK3CA*) were transfected with the *ERBB3* or EGFR siRNAs for 72 hours and cell lysates were analyzed as indicated. Data shown are representative blots of results from 3 independent experiments with similar results. Source data are provided as a Source Data file.

Supplementary Figure 3

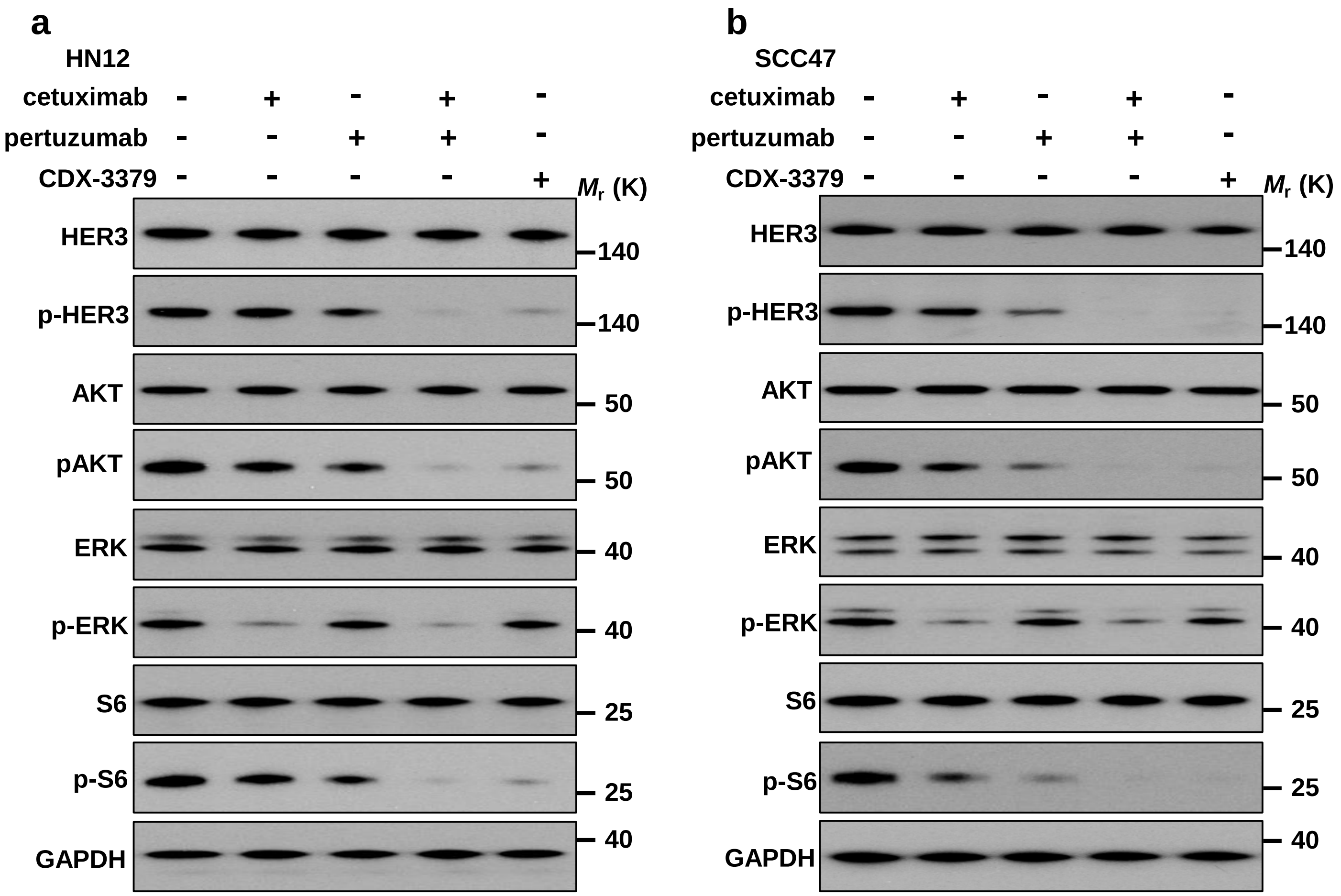

Supplementary Fig. 3. Western blot analysis of signaling events in HNSCC cells treated by cetuximab, pertuzumab, and CDX-3379. (a) HN12 and **(b)** SCC47 cells were serum starved overnight and treated with cetuximab, pertuzumab, cetuximab+pertuzumab, and CDX-3379 (100 ng/ml) for 2 hours. Cell lysates were analyzed as indicated. Data shown are representative blots of results from 3 independent experiments with similar results. Source data are provided as a Source Data file.

Supplementary Figure 4

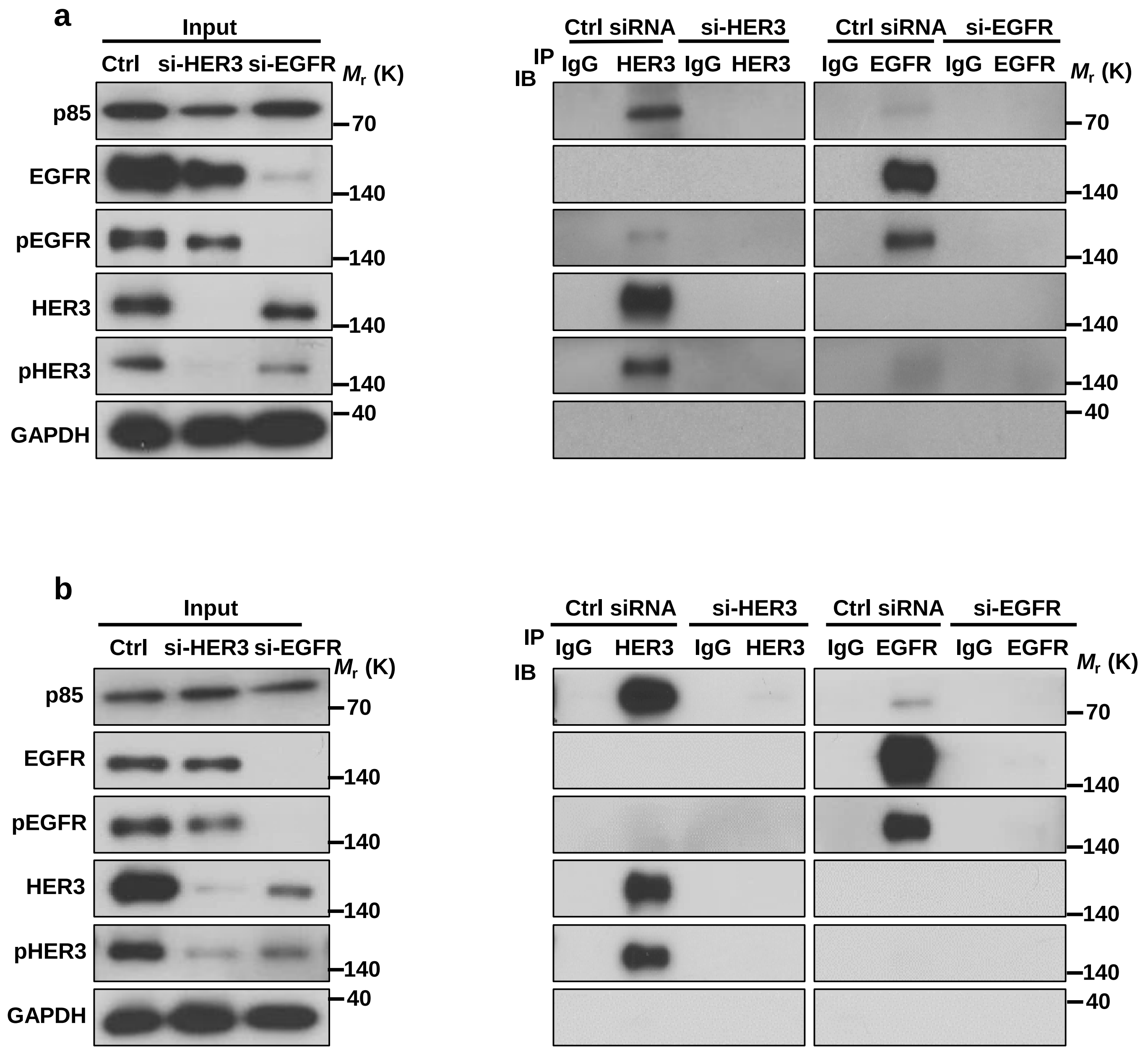

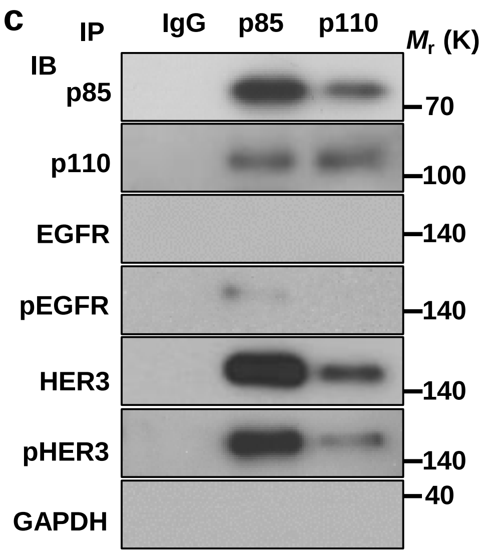

Supplementary Fig. 4. Association between HER3 and EGFR with PI3K in HNSCC cells. (a) Cal27 and **(b)** HN12 cells were transfected with the *ERBB3* or *EGFR* siRNAs for 72 hours, and total cell lysates (input) and HER3 or EGFR immunoprecipitation (IP) were analyzed by western blotting as indicated. **(c)** Analysis of p85 and p110 association with HER3. Cal27 cells were lysed and IP for p85 and p110, and analyzed by western blotting for co-immunoprecipitation (co-IP) with HER3 and EGFR, as indicated. Data shown are representative blots of results from 3 independent experiments with similar results. Source data are provided as a Source Data file.

Supplementary Figure 5

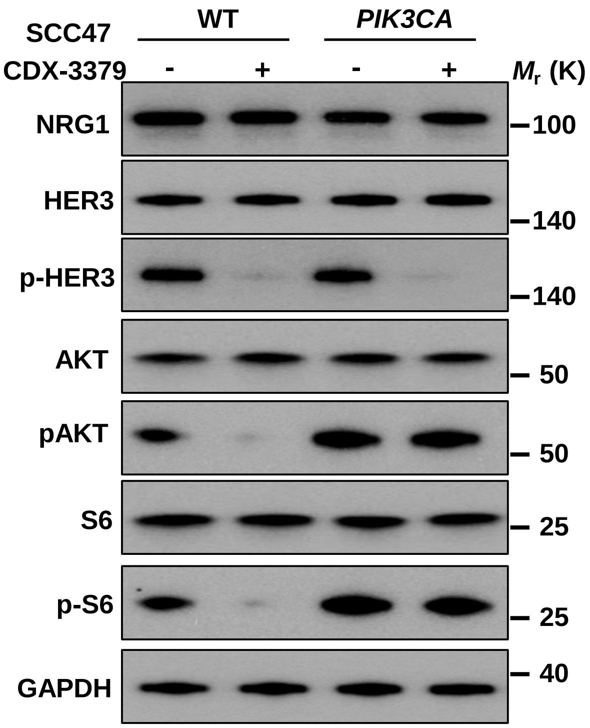

Supplementary Fig. 5. Western blot analysis of signaling events in HNSCC cells treated by CDX-3379. SCC47 wild-type and infected with *PIK3CA* H1047R (*PIK3CA*) of were serum starved overnight and treated by CDX-3379 at 100 ng/ml for 2 hours. Cell lysates were analyzed as indicated. Data shown are representative blots of results from 3 independent experiments with similar results. Source data are provided as a Source Data file.

Supplementary Figure 6

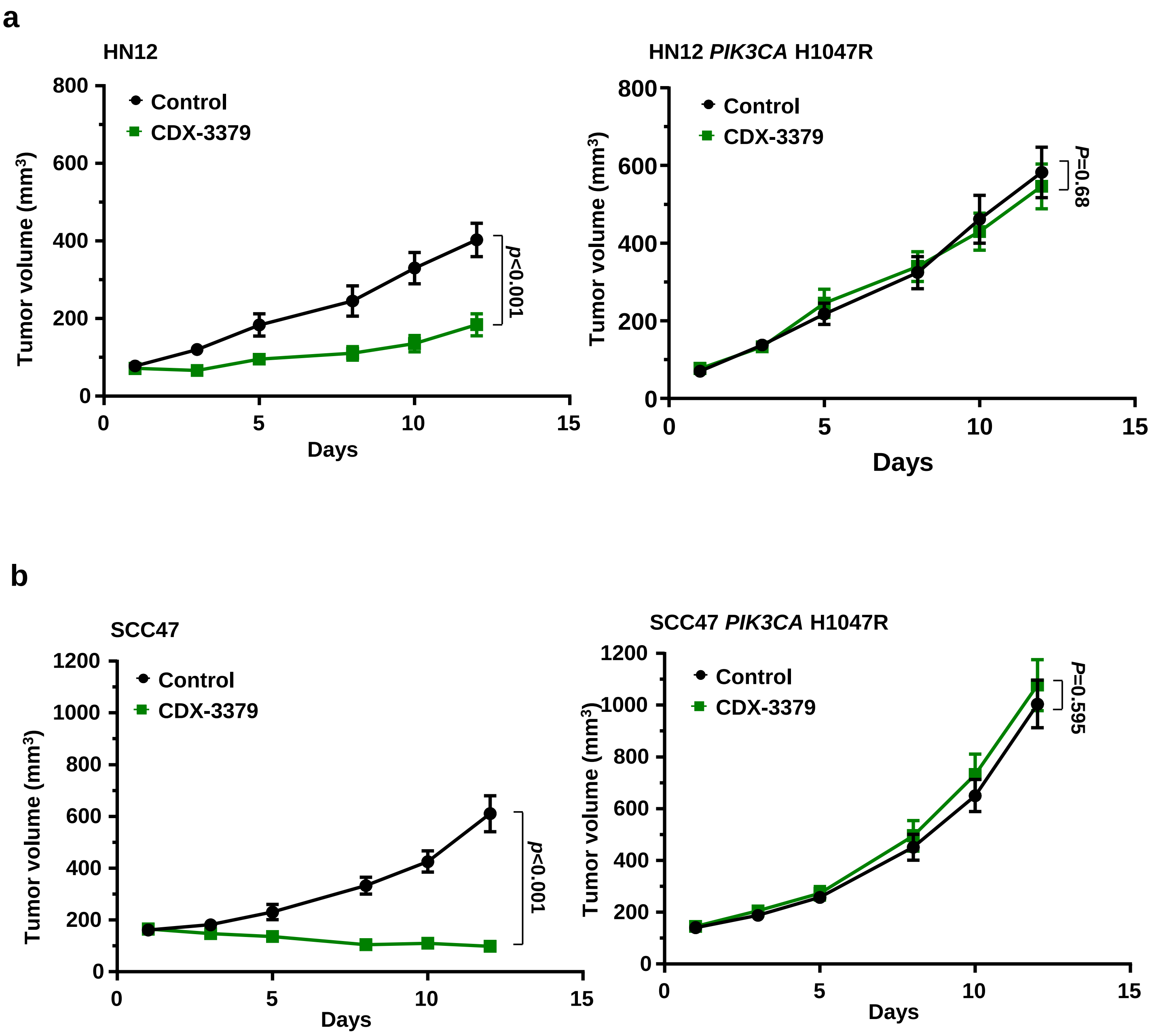

Supplementary Fig. 6. Anti-tumor effect of HER3 inhibition with CDX-3379 antibody in HNSCC. (a) HN12 and **(b)** SCC47 cells wild type and cells expressing *PIK3CA* H1047R were transplanted into the flanks of athymic nude mice, and when they reached around 100-200 mm^3, mice were treated with vehicle diluent or CDX-3379 (10 mg/kg, three times/ week) for the indicated days (n=10 per group). Data are reported as mean ± SEM; two-sided Student's *t*-test. Source data are provided as a Source Data file.

Supplementary Figure 7

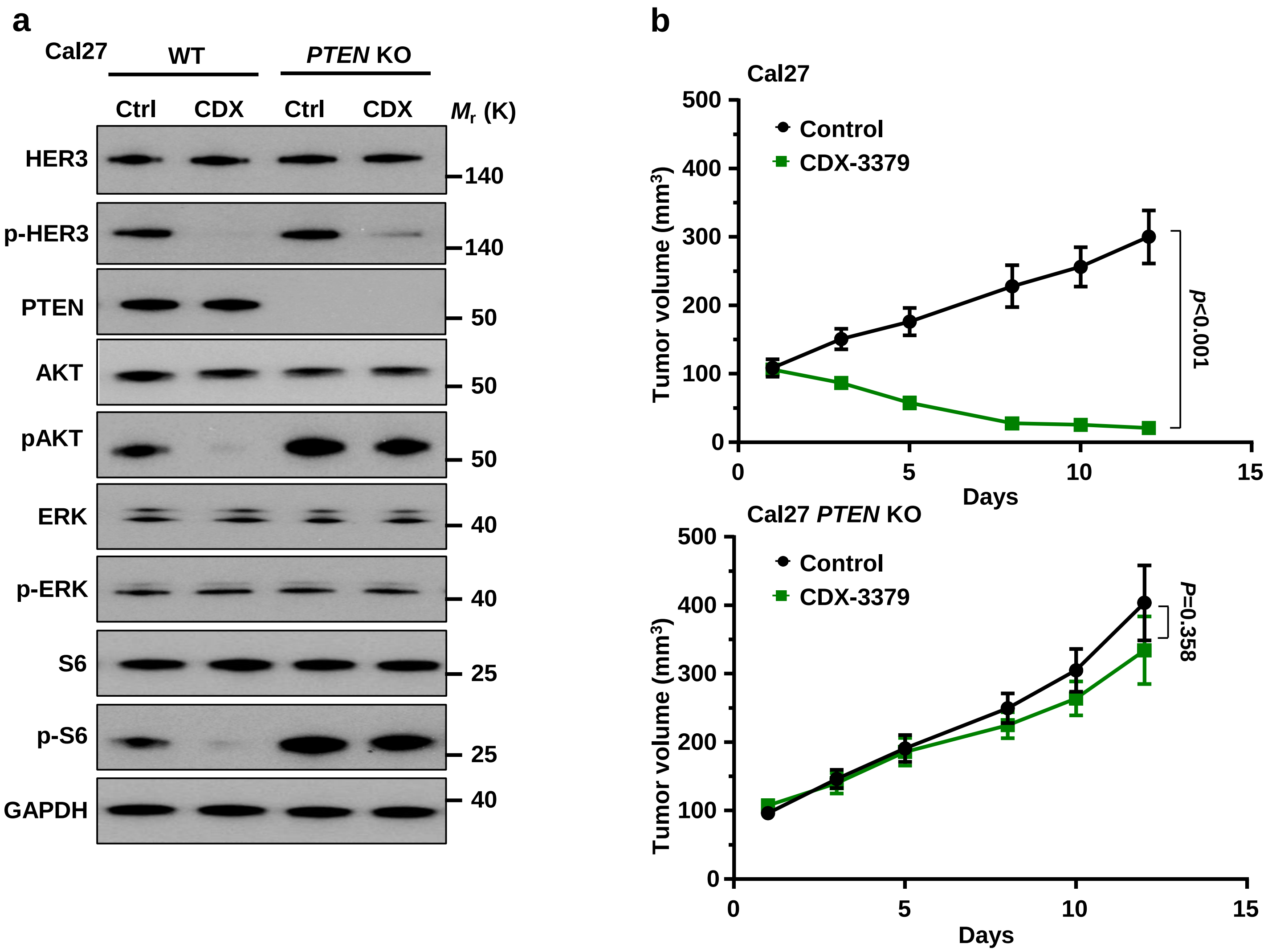

Supplementary Fig. 7. Resistance of CDX-3379 antibody in *PTEN* knock out (KO) HNSCC. (a) Cal27 and Cal27 *PTEN* KO cells were serum starved overnight and treated with CDX-3379 (100 ng/ml) for 2 hours. Cell lysates were analyzed as indicated. Data shown are representative blots of results from 3 independent experiments with similar results. **(b)** Cal27 and Cal27 *PTEN* KO cells were transplanted into the flanks of athymic nude mice, and when they reached around 100 mm^3, mice were treated with vehicle diluent or CDX-3379 (10 mg/kg, three times/ week) for the indicated days (n=10 per group). Data are reported as mean ± SEM; two-sided Student's *t*-test. Source data are provided as a Source Data file.

Supplementary Figure 8

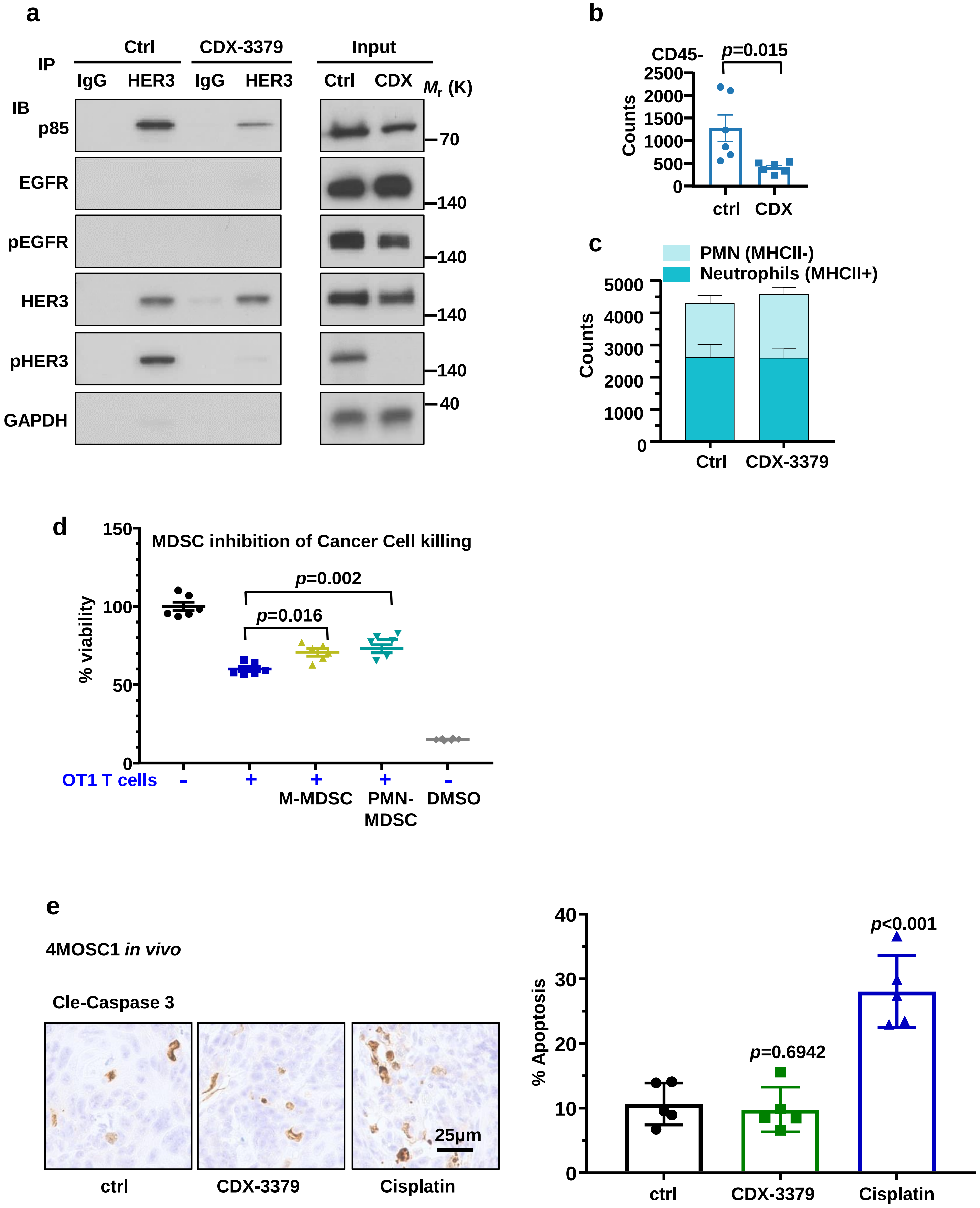

Supplementary Fig. 8. Effect of CDX-3379 on the tumor immune microenvironment in syngeneic HNSCC models. (a) HER3 co-IP analysis of signaling events in 4MOSC1 cells treated by CDX-3379. 4MOSC1 cells were treated by CDX-3379 (1µg/ml) for 1 hour and cell lysates were analyzed as indicated. Data shown are representative blots of results from 3 independent experiments with similar results. **(b)** Counts of CD45- cells from Figure 5d. n=6 per group. Data are reported as mean ± SEM; two-sided Student's *t*-test. **(c)** Counts of PMN-MDSC and Neutrophil cells from figure 5d. n=6 per group. Data are reported as mean ± SEM; PMN-MDSCs are CD45+CD11b+Ly6G+Ly6C^{low}MHC II-, and Neutrophils are CD45+CD11b+Ly6G+Ly6C^{low}MHC II+ **(d)** Myeloid derived suppressor cells isolated from 4MOSC tumors block antigen-specific T cell priming necessary for antitumor cytotoxicity. Splenocytes from OT-1 animals were isolated and stimulated *ex vivo* with aCD3/aCD28 antibodies in the presence of either 5% PMN-MDSCs (Gr-1highLy-6G+) or 5% M-MDSCs (Gr-1dimLy-6G-), isolated from 4MOSC1 tongue tumors with the Miltenyi Biotec bead isolation kit. After a 3-day incubation, stimulated OT-1 splenocytes were harvested and co-cultured with B16-OVA cells for a 48-hour killing assay. The % of viable B16-OVA tumor cells was then measured with the AquaBluer redox indicator, n=6 per group. Data are reported as mean ± SEM; two-sided Student's *t*-test. **(e)** C57Bl/6 mice were implanted with $1x10^6$ 4MOSC1 cells into the tongue. After tumors reached ~30 mm^3, mice were treated IP with of isotype control, CDX-3379 (20 mg/kg), and cisplatin (3 mg/kg) every three days (twice). Left panel, shown are representative immunohistochemical analysis of cle-Caspase 3. Right panel, quantification from images on the left using Qupath software. n=5, Data are reported as mean ± SEM; two-sided Student's *t*-test. Source data are provided as a Source Data file.

Supplementary Figure 9

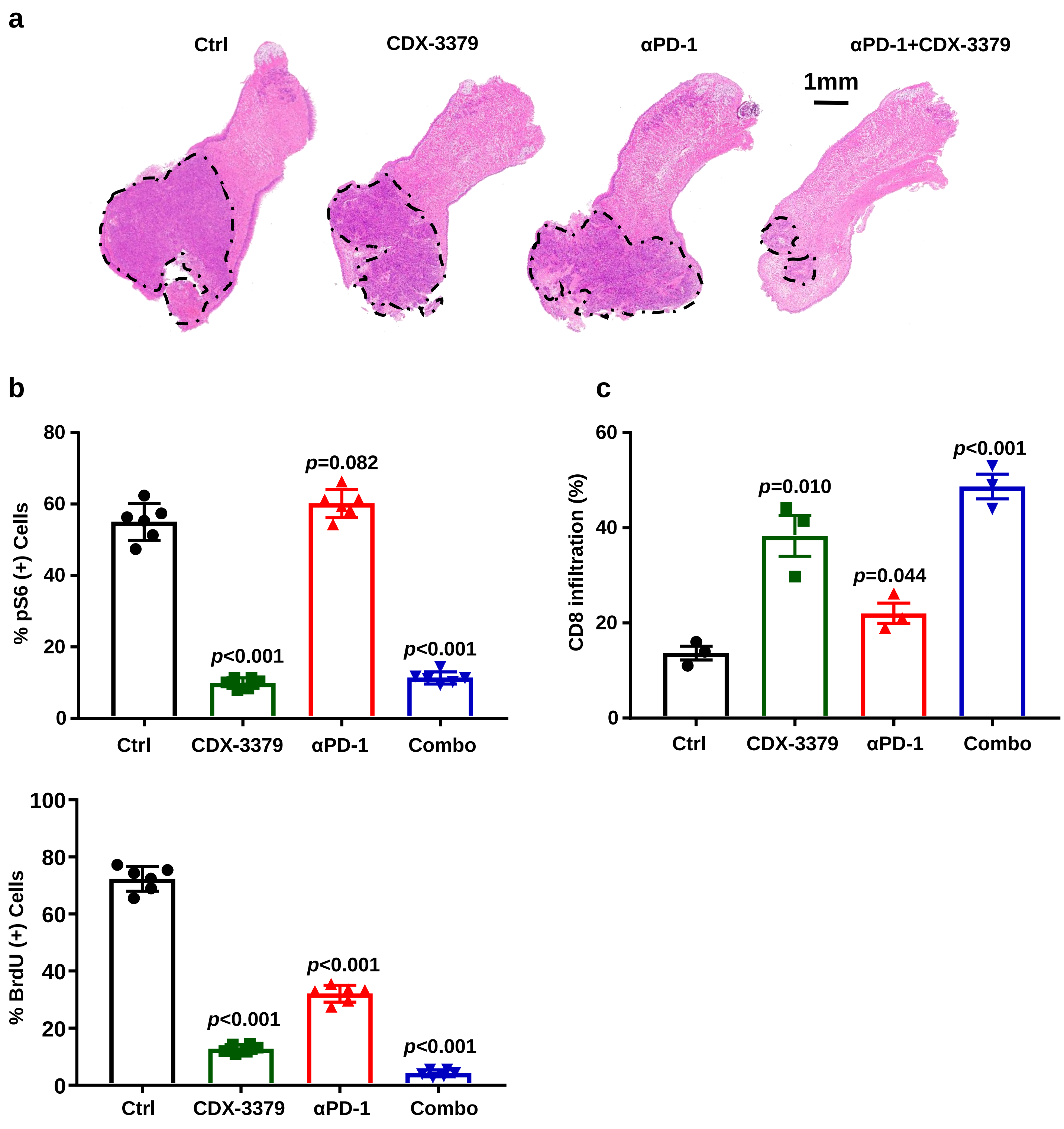

Supplementary Fig. 9. Anti-tumor effect of CDX-3379 combined with PD-1 inhibition in syngeneic HNSCC models. (a) Representative H&E stains of mouse tumors from the experiment in **Fig. 6a** (n = 10 mice per group). **(b)** Automated histological quantification of stainings in **Fig. 6d** (n = 6). **(c)** Quantification of CD8 T cells in **Fig. 6e**. Shown is the average CD8 positivity by three regions of interest (ROI) per mouse, quantified by Qupath software for each condition (n = 3). Data are reported as mean ± SEM; two-sided Student's *t*-test. Source data are provided as a Source Data file.

Supplementary Figure 10

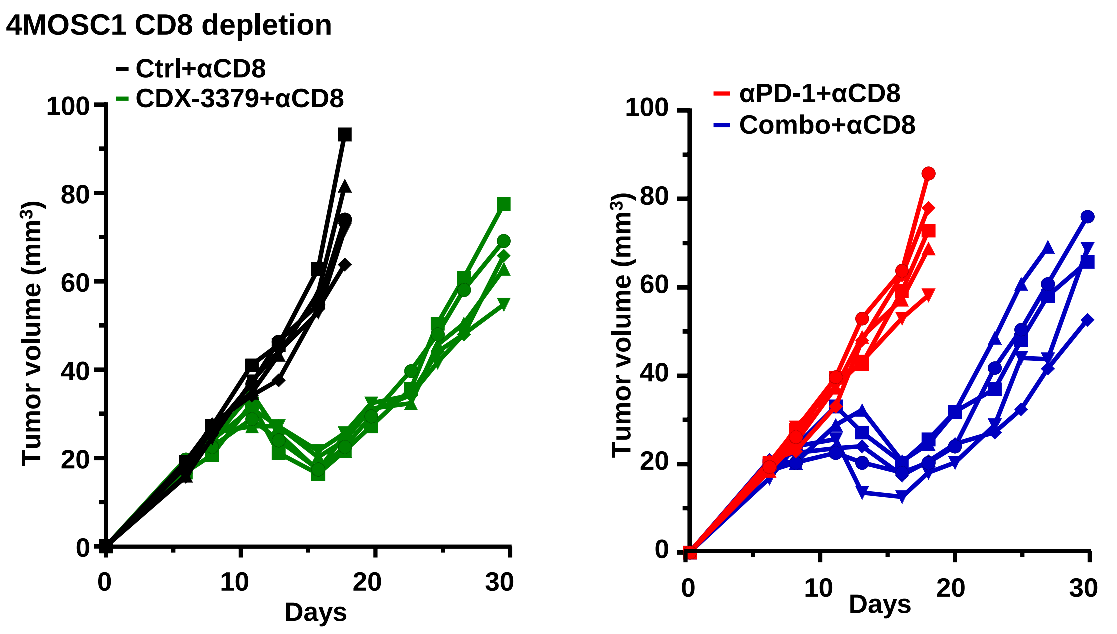

Supplementary Fig. 10. Dependence of anti-HER3 and anti-PD-1 anti-tumor activity on CD8 T cells. Mice were implanted with $1x10^6$ of 4MOSC1 cells into the tongue. After tumors reached ~30 mm^3, C57Bl/6 mice were treated with CD8 T cell depleting antibody daily for 3 days, and treated IP (started the same day of CD8 T cell depletion) with isotype control, CDX-3379 (20 mg/kg), anti-PD-1 (10 mg/kg), or a combination of CDX-3379 and PD-1 three times per week for three weeks (n = 5 per group). Individual growth curves of 4MOSC1 tumor-bearing mice are shown.

Supplementary Figure 11

a

Related to Figure 1e

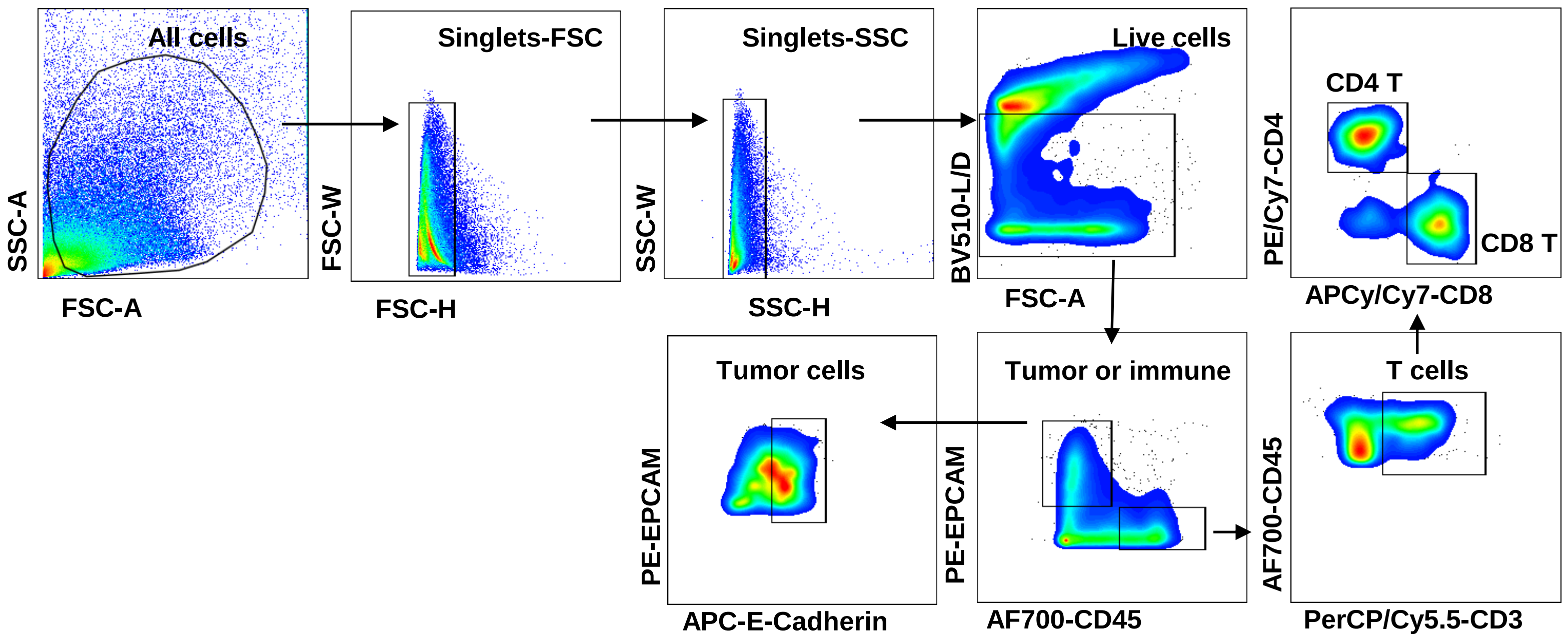

b

Related to Figure 5b, left panel

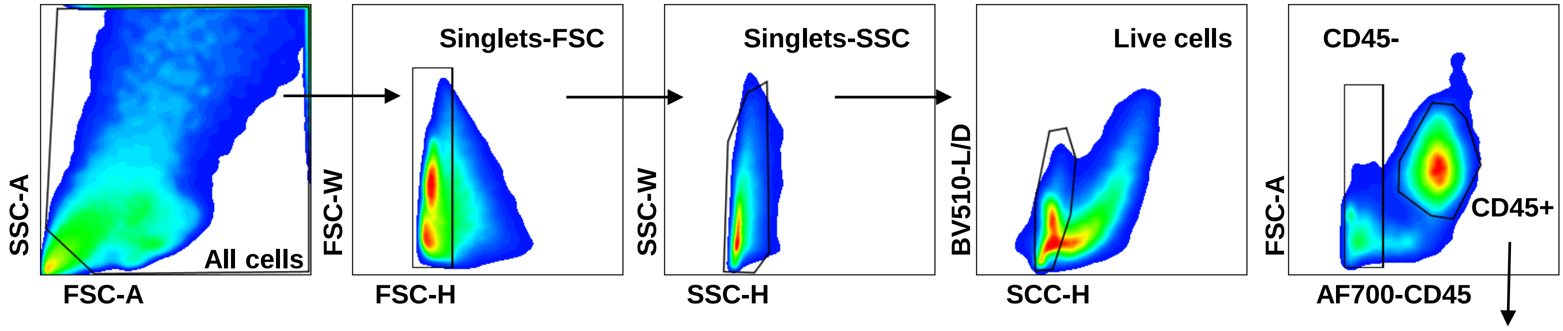

Supplementary Figure 11 continued

c

Related to Figure 5b, right panel

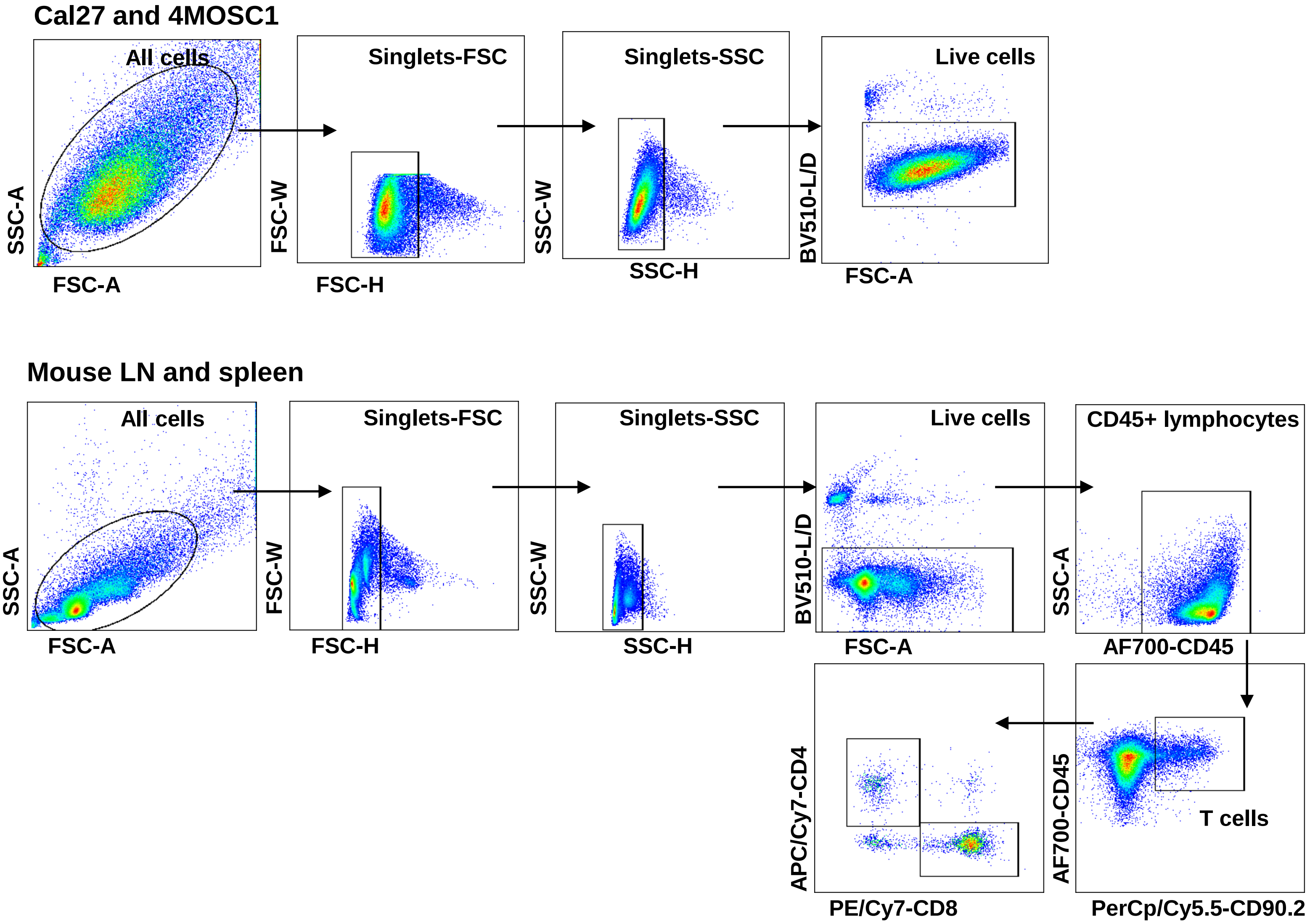

Supplementary Figure 11. Representative flow cytometry gating strategies. Representative flow cytometry plots to quantify mean fluorescence intensity of HER3 in **(a)** related to Figure 1e, **(b)** related to Figure 5b, left panel **(c)** related Figure 5b, right panel
