# Supplementary material for: Disruption of the HER3-PI3K-mTOR oncogenic signaling axis and PD-1 blockade as a multimodal precision immunotherapy in head and neck cancer
Source: Nat Commun. 2021 Apr 22;12:2383. doi: 10.1038/s41467-021-22619-w (PMC8062674; doi:10.1038/s41467-021-22619-w)
Supplement: Supplementary file 2 — Descriptions of Additional Supplementary Files [file 41467_2021_22619_MOESM2_ESM.pdf]

Descritpions of Additional Supplementary Files

Supplementary Data 1
Description: siRNA library screen targeting human kinases in Cal27 cells was conducted to search for genes that affect proliferation of HNSCC cells. Shown are the Z-scores for viability when each gene was knocked down.
