# Supplementary material for: Disruption of the HER3-PI3K-mTOR oncogenic signaling axis and PD-1 blockade as a multimodal precision immunotherapy in head and neck cancer
Source: Nat Commun. 2021 Apr 22;12:2383. doi: 10.1038/s41467-021-22619-w (PMC8062674; doi:10.1038/s41467-021-22619-w)
Supplement: Supplementary file 4 — Reporting Summary [file 41467_2021_22619_MOESM4_ESM.pdf]

nature research

Corresponding author(s): J. Silvio Gutkind, Robert Saddawi-Konefka

Last updated by author(s): Mar 13, 2021

Reporting Summary

Nature Research wishes to improve the reproducibility of the work that we publish. This form provides structure for consistency and transparency in reporting. For further information on Nature Research policies, see our Editorial Policies and the Editorial Policy Checklist.

Statistics

For all statistical analyses, confirm that the following items are present in the figure legend, table legend, main text, or Methods section.

n/a	Confirmed	
☐	☒	The exact sample size (*n*) for each experimental group/condition, given as a discrete number and unit of measurement
☐	☒	A statement on whether measurements were taken from distinct samples or whether the same sample was measured repeatedly
☐	☒	The statistical test(s) used AND whether they are one- or two-sided<br>*Only common tests should be described solely by name; describe more complex techniques in the Methods section.*
☐	☒	A description of all covariates tested
☐	☒	A description of any assumptions or corrections, such as tests of normality and adjustment for multiple comparisons
☐	☒	A full description of the statistical parameters including central tendency (e.g. means) or other basic estimates (e.g. regression coefficient) AND variation (e.g. standard deviation) or associated estimates of uncertainty (e.g. confidence intervals)
☐	☒	For null hypothesis testing, the test statistic (e.g. *F*, *t*, *r*) with confidence intervals, effect sizes, degrees of freedom and *P* value noted<br>*Give P values as exact values whenever suitable.*
☒	☐	For Bayesian analysis, information on the choice of priors and Markov chain Monte Carlo settings
☒	☐	For hierarchical and complex designs, identification of the appropriate level for tests and full reporting of outcomes
☐	☒	Estimates of effect sizes (e.g. Cohen's *d*, Pearson's *r*), indicating how they were calculated

Our web collection on statistics for biologists contains articles on many of the points above.

Software and code

Policy information about availability of computer code

Data collection	TCGA
Data analysis	Qupath (V0.2.0), GraphPad software (V7), FlowJo (V10), Cytobank (V8)

For manuscripts utilizing custom algorithms or software that are central to the research but not yet described in published literature, software must be made available to editors and reviewers. We strongly encourage code deposition in a community repository (e.g. GitHub). See the Nature Research guidelines for submitting code & software for further information.

Data

Policy information about availability of data

All manuscripts must include a data availability statement. This statement should provide the following information, where applicable:

- Accession codes, unique identifiers, or web links for publicly available datasets
- A list of figures that have associated raw data
- A description of any restrictions on data availability

Clinical data used in this study was downloaded from the publicly available cBioPortal (cbioportal.org). A data availability statement is provided in the manuscript text describing data analyzed in this study.

Field-specific reporting

Life sciences study design

All studies must disclose on these points even when the disclosure is negative.

Sample size	Sample sizes were chosen based on the historical data of the variability of tumor growth and treatment response observed and determined to be adequate based on the consistency of measurable differences within and between groups. Additionally, our previous publication was used as a reference: DOI: 10.1038/s41467-019-13471-0
Data exclusions	No data were excluded from these analyses.
Replication	Every experiment was replicated at least three times with near-identical results.
Randomization	Based on the tumor volumes on the first day of treatment, tumor bearing mice were randomly assigned to treatment groups such that each treatment group or time point/treatment group had the same average tumor volume.
Blinding	The data presented did not require the use of blinding. Data reported for mouse experiments were not subjective but rather based on quantitative analyses

Reporting for specific materials, systems and methods

We require information from authors about some types of materials, experimental systems and methods used in many studies. Here, indicate whether each material, system or method listed is relevant to your study. If you are not sure if a list item applies to your research, read the appropriate section before selecting a response.

Materials & experimental systems

n/a	Involved in the study	
☐	☒	Antibodies
☐	☒	Eukaryotic cell lines
☒	☐	Palaeontology and archaeology
☐	☒	Animals and other organisms
☐	☒	Human research participants
☒	☐	Clinical data
☒	☐	Dual use research of concern

Methods

n/a	Involved in the study	
☒	☐	ChIP-seq
☐	☒	Flow cytometry
☒	☐	MRI-based neuroimaging

Antibodies

Antibodies used

All flow cytometry antibodies from BioLegend, San Diego, CA:
For human experiments, we used CD45 (HI30), CD3 (HIT3A), CD8a (HIT8A), CD4 (RPA-T4), Ep-CAM (9CA), E-cadherin (67A4); all antibodies were used at a 1:100 dilution.
For mouse experiments, we used CD45 (30-F11) (1:100), CD90.2 (30-H12) (1:200) , CD8a (53-6.7) (1:100), CD4 (RM4-4) (1:400), Ep-CAM (G8.8) (1:100);
The flow cytometry antibody used for FITC-HER3 detection was obtained from Celldex Therapeutics and used at a 1:100 dilution.
The antibodies for CyTOF are the following:
B220 (RA3-6B2), CD117(2B8), CD11c (N418), CD25 (3C7), CD4 (RM4-5), CD45 (30-F11), CD8a (53-6.7), MHC-II (M5/114.15.2), NKP46 (29A1.4), TCRb (H57-597) are from Fluidigm.
CD103 (2E7), CD115 (AFS98), CD11b (M1/70), CD19 (6D5), CD23 (B3B4), CD3 (145-2C11), CD64 (X54-5/7.1), F4/80 (BM8), FCeRI (Mar-1), FR4 (TH6), Ly6C (HK1.4), Ly6G (1A8), NK1.1 (PK136) are from Biolegend.
Siglec-F (1RNM44N) is from ebioscience; all antibodies were used at a 1:100 dilution.
Therapeutic antibodies from Bio X Cell (West Lebanon, NH, USA): PD-1 antibody (clone J43, catalog #BE0033-2), (isotype antibody (catalog # BE0091) and CD8 depletion antibody (Clone YTS 169.4, catalog #BE0117). CDX3379 is provided by Celldex Therapeutics for both therapeutic purpose and flow cytometry. This antibody is designed to block the activity of ErbB3 (HER3) in both human and mouse.
Immunofluorescence antibodies: CK5 (Fitzgerald, 20R-CP003) (1:500), HER3 (Cell signaling Technology #12708S) (1:200), and CD8 (abcam, ab22378) (1:400).
Immunohistochemistry antibodies: pS6 (Cell Signaling Technology #2211) (1:200), Brdu (Bio-Rad #OBT0030S) (1:100) and Cleaved Caspase-3 (Cell Signaling Technology # 9661) (1:400).
Antibodies for immunoblot analysis and Immunoprecipitation (IP) assay are from Cell Signaling Technology (Danvers, MA), pEGFR (#2234) (1:2000), pHER3 (#4791) (1:2000), p110 (#4255S, 1:2000) pAKTT308 (#2965, 1:3000), pS6 (#2211) (1:5000), S6 (#2217) (1:5000), pERK (#4370) (1:2000), ERK (#9106) (1:3000) and GAPDH (# 2118) (1:8000), or Santa Cruz Biotechnology (Dallas, TX), EGFR (#sc-120) (1:2000), HER3 (#sc-7390) (1:2000), or Millipore-Sigma (Billerica, MA), p85 (# ABS234) (1:1000).

Validation

All antibodies were validated by the supplier and were checked in the lab by comparing to the manufacturer's or in-house results. All antibodies were validated by the supplier and were checked in the lab by comparing to the manufacturer's or in-house results.
Statement from BioLegend: BioLegend antibodies undergo an extensive series of testing to ensure quality at every step in the

manufacturing process, as well as maintaining quality after the sale. Statement from Bio X Cell: Our InVivoPlus™ antibodies feature all the great qualities of our InVivoMab™ antibodies. The InVivoPlus™ versions of our products are structurally and functionally identical to the InVivoMab™ versions with the added benefit of additional QC measures. InVivoPlus™ antibodies are screened for murine pathogens using ultrasensitive qPCR, screened for protein aggregation via dynamic light scattering, feature advanced binding validation via flow cytometry, ELISA, and/or Western blot, and are guaranteed to contain less than 1 endotoxin unit per milligram. Our InVivoPlus™ line of antibodies are designed to exceed the strict demands and rigorous standards required for in vivo work at any research organization. For Fluidigm, please see antibody information from this link: https://www.fluidigm.com/citations/antibody-validation, and antibody titration information from this link: https://www.fluidigm.com/citations/titration-of-mass-cytometry-reagents. For CK5 (Fitzgerald, 20R-CP003) , please see the information in this link, https://www.fitzgerald-fii.com/cytokeratin-5-antibody-20r-cp003.html. And from the literature, this antibody has been used in the following: PLoS ONE on 20 June 2019 by Beeler, J. S., Marshall, C. B., et al.p73 regulates epidermal wound healing and induced keratinocyte programming. And The Journal of Biological Chemistry on 17 July 2015 by Huang, Y., Hamana, T., et al. Prostate Sphere-forming Stem Cells Are Derived from the P63-expressing Basal Compartment. Statement from abcam: Antibody specificity is confirmed by looking at cells that either do or do not express the target protein within the same tissue. Initially, our scientists will review the available literature to determine the best cell lines and tissues to use for validation. We then check the protein expression by IHC/ICC to see if it has the expected cellular localization (Figure 3). If the localization of the signal is as expected, this antibody will pass and is considered suitable for use in IHC/ICC. We use a variety of methods, including staining multi-normal human tissue microarrays (TMAs), multi-tumor human TMAs, and rat or mouse TMAs during antibody development. These high-throughput arrays allow us to check many tissues at the same time, providing uniformly as all tissues are exposed to the exact same conditions. Statement from Cell Signaling Technology: https://www.cellsignal.com/contents/our-approach-antibody-validation-principles/antibody-validation-for-immunofluorescence/ourapproach-validation-if . Statement from BioRad: Bio-Rad conducts rigorous in house testing to guarantee that all of our antibodies meet our internal benchmarks and perform in their designated applications as expected. Our stringent quality control process is recognized by ISO9001:2015 certification at our manufacturing sites in Kidlington, Oxfordshire, UK, and Puchheim, Germany.
We clearly display recommended applications for every antibody on our website and product datasheet. In addition, Bio-Rad Quality and Performance Guarantee ensures that if a catalog antibody batch doesn't meet the specifications as described on the datasheet, and the issue cannot be resolved by our technical support team, we will replace the antibody free of charge or offer a credit.
Statement from ThermoFisher Scientific: Thermo Fisher Scientific is committed to adopting validation standards for our Invitrogen antibody portfolio. The Advanced Verification badge is applied to products that have passed application and specificity testing. This badge can be found in the search results and at the top of the product specific webpages. Data supporting the Advanced Verification badges can be found in product specific data galleries.

Eukaryotic cell lines

Policy information about cell lines

Cell line source(s)

4MSOC1 syngeneic cell lines were generated in-house and manipulated according to our previous publicatoin: DOI: 10.1038/s41467-019-13471-0. MOC1 cells were generously provided by Dr. R. Uppaluri lab. Human HNSCC cell lines Cal27, HN12, SCC47 and Detroit 562 were obtained from the NIDCR (National Institute of Dental and Craniofacial Research) cell collection.

Authentication

DNA authentication of cell lines was confirmed by multiplex STR profiling (Genetica DNA Laboratories, Inc. Burlington, NC) to ensure the consistency of cell identity.

Mycoplasma contamination

All cell lines are frequently tested for mycoplasma contamination. No presence of mycoplasma was found according to Mycoplasma Detection Kit-QuickTest from Biomake (Houston, TX, USA).

Commonly misidentified lines
(See ICLAC register)

No commonly misidentified cells were used.

Animals and other organisms

Policy information about studies involving animals; ARRIVE guidelines recommended for reporting animal research

Laboratory animals

Female athymic mice and C57Bl/6 mice (4–6 weeks of age and weighing 16–18g) were purchased from Charles River Laboratories (Worcester, MA, USA). All the animal studies using HNSCC tumor xenografts and oral carcinogenesis studies were approved by the Institutional Animal Care and Use Committee (IACUC) of University of California, San Diego, with protocol ASP #S15195. Animal housing conditions are described in the manuscript.

Wild animals

Study did not involve wild animals.

Field-collected samples

Study did not involve samples from the field.

Ethics oversight

All studies were approved by the Institutional Animal Care and Use Committee (IACUC) of University of California, San Diego, and mouse procedures were performed following ACP guidelines.

Note that full information on the approval of the study protocol must also be provided in the manuscript.

Human research participants

Policy information about studies involving human research participants

Population characteristics

The relevant covariate characteristics for the surgical specimen are as follows: the data presented in Figure 1 E and 1F are from a representative fresh surgical specimen of a Stage II T2N0M0 primary tongue squamous cell carcinoma. This particular experiment examined the expression of HER3 within the tumor and immune compartments in the tumor-microenvironment; and, in this context, additional covariate population characteristics are not relevant. This information also appears in legend

	for Figure 1.
Recruitment	The choice to closely examine HER3 expression was made by intention in an early stage human oral cavity cancer, as our preclinical model reflects such a human cancer. Figure 3D which interrogates TCGA data more comprehensively examines HER3 expression across a broad swath of human head and neck squamous cell carcinomas. Together, these approaches reduce any inherent sampling bias.
Ethics oversight	Specimen samples were provided by Moores Cancer Center at UC San Diego Health Comprehensive Biorepository, which is funded by the National Cancer Institute (NCI P30CA23100). The BTTR policies and procedures were written in accordance with federal policy on the Protection of Human Subjects (DHHS Policy 45 CFR Part 46, FDA Policy 21 CFR Parts 50 and 56). We have obtained informed consent from all participants. The data consist of time of collection, diagnosis of the tumor stage and site, clinical outcome and other demographical information. Patient information is deidentified as much as possible and the data are safeguarded with multiple levels and layers of security. Encryption is required for all health-related personal data. Human surgical specimens were used for histological and immunofluorescence evaluation and flow cytometric analysis.

Note that full information on the approval of the study protocol must also be provided in the manuscript.

Flow Cytometry

Plots

Confirm that:

- [x] The axis labels state the marker and fluorochrome used (e.g. CD4-FITC).
- [x] The axis scales are clearly visible. Include numbers along axes only for bottom left plot of group (a 'group' is an analysis of identical markers).
- [x] All plots are contour plots with outliers or pseudocolor plots.
- [x] A numerical value for number of cells or percentage (with statistics) is provided.

Methodology

Sample preparation	Tumors were dissected, minced, and re-suspended in complete media (DMEM with 10% FBS and 1% antibiotics) supplemented with Collagenase-D (1mg/mL; Roche) and incubated at 37°C for 30 minutes with shaking to form a single-cell suspension. Tissue suspensions were washed with fresh media and passed through a 100-µm strainer.
Instrument	BD LSRFortessa
Software	FlowJo
Cell population abundance	Cells were not sorted in this study.
Gating strategy	Single cells were gated from FSC/SSC (height/width) and live/dead cells were discriminated using BD HorizonTM Fixable Viability Stain 510. Cells were further characterized according to the following characteristics: cytotoxic T cells (CD45+CD3+CD8+), helper T cells (CD45+CD3+CD4+), and epithelial cells (Ep-CAM+ E-cadherin+).

- [x] Tick this box to confirm that a figure exemplifying the gating strategy is provided in the Supplementary Information.
